# A Canadian survey of medical students and undergraduate deans on the management of patients living with obesity

**DOI:** 10.1186/s12909-022-03636-9

**Published:** 2022-07-21

**Authors:** Nathan J. Katz, Olivia Lovrics, Boris Zevin

**Affiliations:** 1 School of Medicine, Office of Professional Development and Educational Scholarship, Queen’s University, Kingston, ON Canada; 2 Division of General Surgery, Department of Surgery, McMaster University, Hamilton, ON Canada; 3 Department of Surgery, Kingston General Hospital, Queen’s University, 76 Stuart Street, Kingston, ON K7L 2V7 Canada

**Keywords:** Obesity, Medical education, Medical student, Curriculum, Survey

## Abstract

**Background:**

With over 26% of Canadian adults living with obesity, undergraduate medical education (UGME) should prepare medical students to manage this chronic disease. It is currently unknown how the management of patients living with obesity is taught within UGME curricula in Canada. This study (1) examined the knowledge and self-reported competence of final-year medical students in managing patients living with obesity, and (2) explored how this topic is taught within UGME curricula in Canada.

**Methods:**

We distributed two online surveys: one to final-year medical students, and another to UGME deans at 9 English-speaking medical schools in Canada. The medical student survey assessed students’ knowledge and self-reported competence in managing patients living with obesity. The dean’s survey assessed how management of patients living with obesity is taught within the UGME curriculum.

**Results:**

One hundred thirty-three (6.9%) and 180 (9.3%) out of 1936 eligible students completed the knowledge and self-reported competence parts of the survey, respectively. Mean knowledge score was 10.5 (2.1) out of 18. Students had greatest knowledge about etiology of obesity and goals of treatment, and poorest knowledge about physiology and maintenance of weight loss. Mean self-reported competence score was 2.5 (0.86) out of 4. Students felt most competent assessing diet for unhealthy behaviors and calculating body mass index. Five (56%) out of 9 deans completed the survey. A mean of 14.6 (5.0) curricular hours were spent on teaching management of patients living with obesity. Nutrition and bariatric surgery were most frequently covered topics, with education delivered most often via large-group sessions and clinical activities.

**Conclusions:**

Canadian medical students lack adequate knowledge and feel inadequately prepared to manage patients living with obesity. Changes to UGME curricula may help address this gap in education.

**Supplementary Information:**

The online version contains supplementary material available at 10.1186/s12909-022-03636-9.

## Background

Obesity, defined as a body mass index (BMI) ≥ 30 kg/m^2^, is a public health concern in Canada [1, 2]. Common obesity-related comorbidities such as hypertension, type II diabetes, and cancer, decrease quality of life and increase mortality [3, 4]. Patients living with obesity face high-levels of weight-bias and stigmatization in healthcare settings, creating systemic barriers that lead to poorer health outcomes [5, 6]. Addressing obesity management by physicians has been shown to improve health outcomes of patients living with obesity [7]. Despite rising rates of obesity in Canada, physicians in primary-care feel poorly prepared to manage patients living with obesity [8, 9]. This problem, however, is not unique to primary care; physicians in various specialties also report similar challenges, suggesting a knowledge-gap at the undergraduate medical education (UGME) level [10–14]. In light of several factors – this possible knowledge-gap, the greater opportunity for standardization of educational content and delivery that UGME affords, and the relevance of this topic to all physicians regardless of practice – it is plausible that educational content regarding management of patients living with obesity may be best delivered during UGME, rather than being reserved for postgraduate medical education or continuing professional development. In fact, there is consensus in the literature that medical students should be taught how to manage patients living with obesity; which includes being able to discuss behavioural, pharmacological, and surgical treatment options with patients [14–21]. However, there is a lack of understanding on how this topic is currently being taught at the UGME level in Canada and whether graduating students are competent in managing patients living with obesity [22–24]. A representative survey of American medical schools conducted in 2020 demonstrated a paucity of formal education around management of patients living with obesity; reporting consistently low prioritization to develop obesity curricula, and little to no teaching on fundamental obesity topics (such as nutrition, behavioral, and physical activity interventions) among one-third of schools [25]. The authors reported that an overburdened curriculum was the most commonly cited barrier to implementing obesity education, and they suggested using obesity-related competencies developed by recent initiatives such as OMEC (Obesity Medicine Education Collaborative) to guide educators seeking to design obesity curricula and evaluate obesity knowledge [25]. Similarly, a 2018 Norwegian study found obesity-related knowledge to be inadequate in Norwegian medical students, most notably in the areas of physiology, diagnosis, long-term treatment options, and weight management [26].

Our objectives with this study were to: (1) examine the knowledge and self-reported competence of final-year Canadian medical students in managing patients living with obesity, and (2) explore how management of patients living with obesity is currently taught within the UGME curricula in Canada.

## Methods

### Subjects

We recruited final-year medical students and UGME deans from English-speaking medical schools in Canada. We defined final-year medical students as students who were graduating at the end of the academic year when the survey was distributed. We defined the UGME deans as the dean and/or their delegate.

### Study design

We administered a cross-sectional survey between August 2019 and November 2020. Surveys were created and disseminated using Qualtrics (Qualtrics, Provo, UT). The study was approved by our institution’s Health Sciences & Affiliated Teaching Hospitals Research Ethics Board, and all methods were performed in accordance with the relevant guidelines and regulations (see Additional file 1 for complete study protocol, and Additional file 2 for the Checklist for Reporting Results of Internet E-Surveys (CHERRIES)).

### Survey design

#### Medical students’ survey

We designed this survey in two parts. Part one examined medical students’ knowledge and part two examined self-reported competence in managing patients living with obesity. For the knowledge part of the survey, we used an 18-item multiple-choice style questionnaire from a 2018 study by Martins and Norsett-Carr [26]. Questions were grouped into 8 domains: Physiology, Etiology, Diagnosis, Goals for Obesity treatment, Conservative Treatment of Obesity, Surgery, Consequences of Obesity, and Weight-Loss Maintenance (see Additional file 3). For the competence part of the survey, we used a 15-item questionnaire from Jay et al. [13] to explore the self-reported competence of students in managing patients living with obesity. This questionnaire used a 5As construct to explore students’ ability to Assess, Advise, Agree, Assist, and Arrange management of patients living with obesity (see Additional file 4). Each self-reported competency was measured on a 4-point scale: 1) “Know very little about and not able to perform”; 2) “Know something about and somewhat able to perform”; 3) “Able to perform well”; 4) “Able to teach others how to perform”.

We offered medical students, who participated in the study, the option to request personalized feedback on their answers for the knowledge part of the survey. This was done after survey completion to provide students with an opportunity for self-reflection and learning. The personalized feedback included evidence-based explanations for each correct and incorrect answer (see Additional file 5).

We piloted the survey with five final-year medical students at our institution in July of 2019. We did not collect demographic data, such as the name of medical school or the length of their medical program, to ensure anonymity of participants’ responses.

#### UGME deans’ survey

We designed the UGME dean’s survey to explore how management of patients living with obesity is currently taught within the UGME curriculum of each school. The survey included 7-items that explored (1) number of course hours dedicated to the teaching of management of patients living with obesity; (2) modalities used to teach this content (e.g., lecture, group learning, community-based learning); and (3) presence of any programs with a specific focus on the management of patients living with obesity (see Additional file 6). We piloted the dean’s survey at our institution with the local UGME deans’ office.

### Survey distribution and data collection

We contacted the UGME offices of the 14 English-based medical schools in Canada inviting them to participate in our study. Nine out of 14 medical schools agreed to proceed with the local research ethics board (REB) application to allow medical students from that school to participate in our study. One school rejected our request for participation, and 4 schools did not respond to our multiple requests for participation. Once the REB approval at each medical school was granted, we distributed the survey electronically to all final-year medical students in that school and to their UGME dean’s office. We embedded the letter of information and consent material at the start of the survey. Participation was voluntary; however, we offered one $200 CAD lottery prize to medical students as incentive to participate.

We also sent an invitation to participate in the study directly to medical students in Ontario, Canada, via an email from the Ontario Medical Students Association on October 10, 2020. The Ontario Medical Students Association has student representatives from 6 English-speaking schools in Canada. This invitation aimed to recruit students who had not had a chance to participate.

### Data analysis

We scored the medical students’ survey out of 18 points for the questions assessing knowledge (minimum score 0, maximum score 18), and out of 60 points for the questions assessing self-reported competence (15 questions, each scored 1, 2, 3, or 4; minimum score 15, maximum score 60). We performed statistical analysis in Excel 2007. We conducted a Spearman’s rank-order correlation to explore whether there was an association between students’ knowledge scores and self-reported competence scores. We report descriptive statistics as number and percentage (categorical variables) or as mean and standard deviation (continuous variables). We used the narrative comments from the dean’s survey to inform our discussion.

## Results

### Medical students’ survey

A total of 207 out of 1936 (10.7%) medical students across 9 English-speaking Canadian medical schools agreed to participate in the study. Of these, 180 students (9.3%) completed the competence part of the survey, and 133 students (6.9%) completed both knowledge and competence parts of the survey. Twenty-seven out of 207 students started the survey but did not answer any questions.

#### Knowledge regarding management of patients living with obesity

**Fig. 1:**
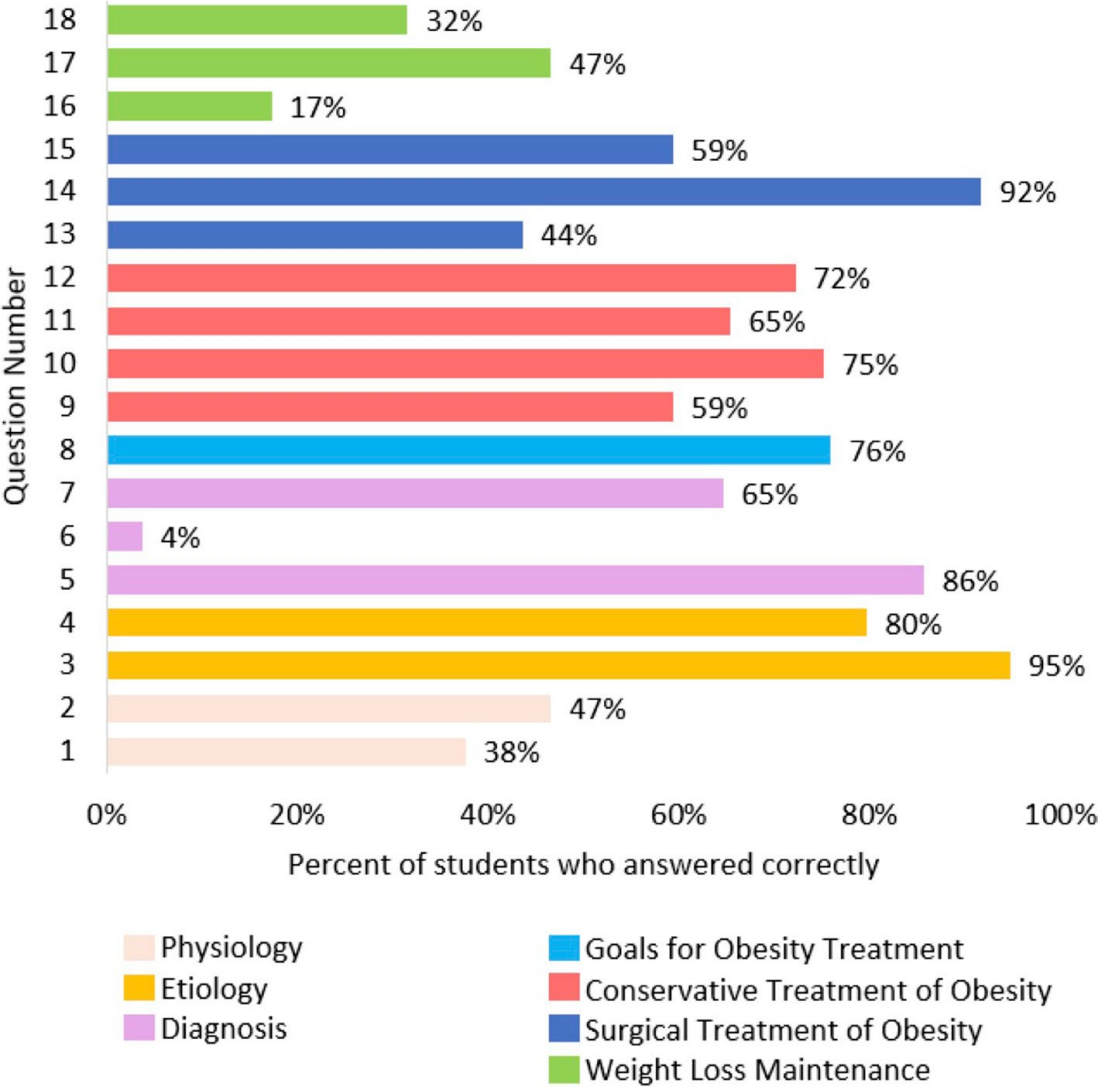
Medical student knowledge of obesity management scores by individual question, grouped by knowledge domain (*n* = 133). Questions adapted from Obesity Knowledge among Final-Year medical Students in Norway, by Martins and Norsett-Carr (2017) [26]

**Fig. 2:**
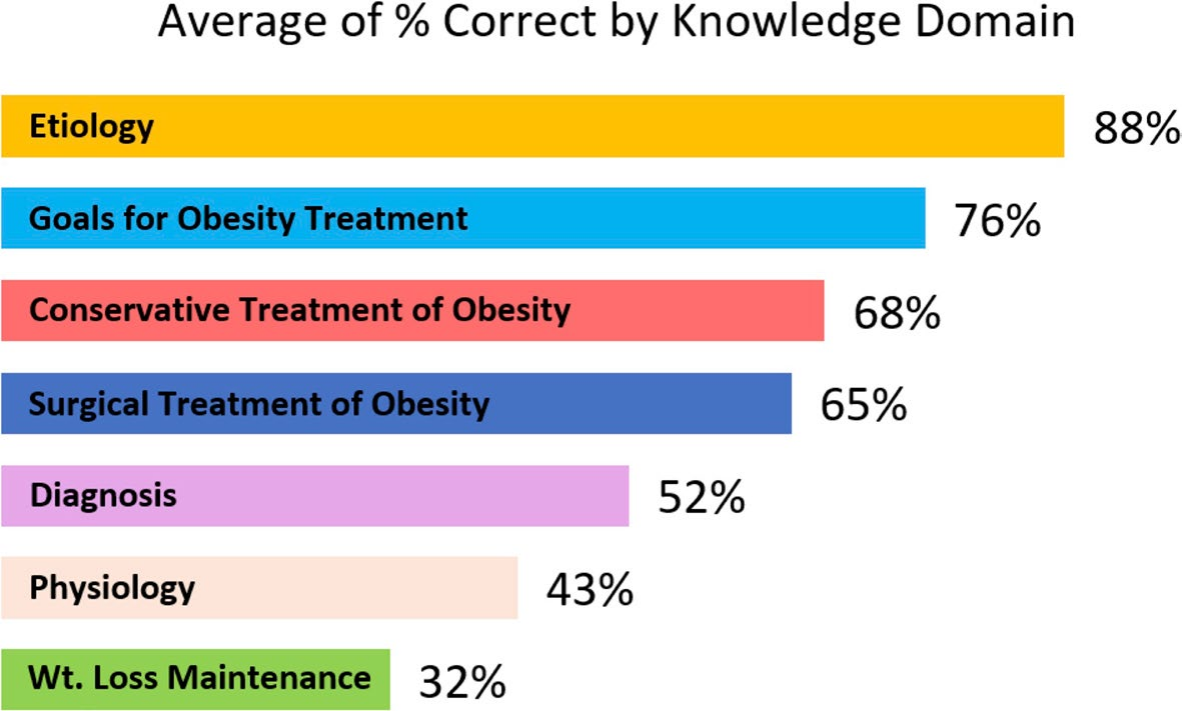
Medical student knowledge of obesity management scores averaged by knowledge domain (*n* = 133). Questions adapted from Obesity Knowledge among Final-Year medical Students in Norway, by Martins and Norsett-Carr (2017) [26]

Mean score for knowledge was 10.5 out of 18 (58%, SD = 2.1, *n* = 133). The number of students with correct responses for each question is reported in Fig. 1. Average correct answers by knowledge domain are reported in Fig. 2. A total of 106 out of 133 (80%) students opted to receive the personalized feedback for their answers.

#### Self-reported competence in managing patients living with obesity

**Fig. 3:**
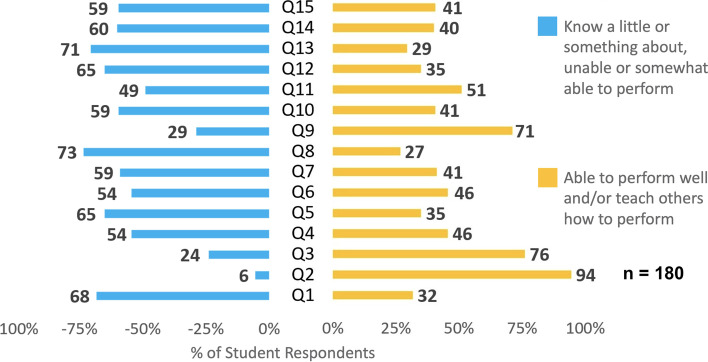
Medical Student Self-Assessed Competencies (*n* = 180). Question begins with “I can…” followed by the stem below. Some stems have been condensed for presentation here. The full survey may be found in Additional file 4. Q1: Use 24-hour recall, food record, or food frequency to obtain diet history; Q2: Determine body mass index (BMI); Q3: Assess diet for unhealthy behaviours associated with obesity; Q4: Ascertain each patient’s readiness and ability to work on weight loss; Q5: Recognize and screen for common psychosocial comorbidities in those with obesity; Q6: Perform targeted history and physical examination to identify common obesity-related comorbidities; Q7: Discuss risks associated with obesity; Q8: Respond to a patient’s questions regarding treatment options; Q9: Assess level of physical activity and provide guidance; Q10: Assist in goal setting related to permanent lifestyle changes; Q11: Prescribe plan for exercise / physical activity; Q12: Use motivational interviewing to change behaviour; Q13: Provide counselling intervention regarding weight loss; Q14: Recognize and refer patients with eating disorders; Q15: Collaborate with and refer to allied health

The overall mean competence score was 2.5 (0.9) out of 4 for the 180 students who responded to this part of the survey. Mean scores for each question are listed in Table 1. Students rated themselves as 3 or above (“able to perform well” or “able to teach others how to perform”) only on two competencies: “Determining the body mass index (BMI) from weight and height measurements” (M = 3.59, SD = 0.63) and “Assessing diet for common unhealthy behaviors associated with obesity” (M = 3.00, SD = 0.74). For the remaining 13 out of 15 competencies, students rated themselves as “knowing something about” or “knowing very little about.” Notably, for the competency of “Responding to a patient’s questions regarding treatment options including behavior change, medications, and surgery” only 4 students (2%) rated themselves as “able to teach others how to perform” and only 44 students (24%) rated themselves as “able to perform well” (see Fig. 3).

**Table 1:** Medical student self-perceived competence questionnaire

Qu #	Questions. I can…	M (SD)
2	Determine body mass index (BMI) from weight and height measurements	3.59 (0.63)
3	Assess diet for common unhealthy behaviours associated with obesity (e.g. sweetened beverages, nutritional quality of snacks, frequent meals from fast food restaurants, etc.)	3.00 (0.74)
9	Assess current level of physical activity and provide guidance for setting physical activity goals for optimal health	2.90 (0.79)
11	Prescribe plan for exercise / physical activity	2.60 (0.87)
4	Ascertain each patient’s readiness and ability to work on weight loss according to health beliefs and stage of change	2.52 (0.76)
6	Take a targeted history and conduct a physical examination to identify common co-morbidities (e.g. arthritis, diabetes, PCOS…)	2.44 (0.76)
7	Discuss the effect of obesity on present and future health and personalize risk to each patient	2.40 (0.74)
10	Assist patient in setting realistic goals for weight loss based on making permanent lifestyle changes	2.38 (0.82)
14	Recognize and refer patients with eating disorders	2.34 (0.73)
5	Recognize and screen for common psychosocial problems in obese patients including depression, emotional eating, and binge eating	2.34 (0.77)
15	Collaborate with registered dieticians and refer to community nutrition resources when appropriate	2.34 (0.84)
12	Use motivational interviewing to change behaviour	2.32 (0.83)
1	Use 24-hour recall, food record, or food frequency to obtain diet history	2.24 (0.83)
13	Provide brief counseling intervention to help patient lose weight	2.18 (0.81)
8	Respond to a patient’s questions regarding treatment options including behaviour change, medications, and surgery	2.11 (0.71)

Scores show Mean (M) and Standard Deviation (SD) for all responses (*n* = 180). Questions were scored on a 1–4 likert scale: 1 – “Know very little about and not able to perform”; 2 – “Know something about and somewhat able to perform”; 3 - “Able to perform well”; 4 – “Able to teach others how to perform”. Questionnaire adapted from Do Internists, Pediatricians, and Psychiatrists Feel Competent in Obesity Care?, by Jay et al. (2008) [13]

Two questions focused on examining student’s self-reported competence in assessing a patient’s diet. Question 1 referred to using a standardized 24-hour food frequency questionnaire (FFQ), and Question 3 explored the topic of diet more broadly by asking students about their ability to assess for common unhealthy behaviors associated with obesity. Thirty two percent of students reported being “able to perform well” and/or “teach others how to perform” the FFQ, while 76% of students reported being “able to perform well” and/or “teach others how to perform” an assessment of diet for unhealthy behaviors.

There was no significant correlation between students’ knowledge and self-reported competence in managing patients living with obesity (r_s_(131) = 0.027, *p* = 0.754).

### UGME dean’s survey

A total of 8 of 14 UGME deans’ offices agreed to participate in the survey; however, only 5 (36%) completed the survey (see Table 2). Four of 5 schools provided an estimate for the average number of curricular hours in undergraduate medical education dedicated to teaching students about the physiology, diagnosis, and management of patients living with obesity; the average of these values was 14.6 (SD = 5.0) hours over 3–4 years of medical school. All schools reported using multiple instructional modalities to deliver this content across at least two school years. Only one school used a non-traditional teaching method, which they described as a half-day nutrition and obesity program conducted at a grocery store by a chef and Registered Dietitian (School 1). No schools offered a dedicated clerkship rotation focused on the management of patients living with obesity.

**Table 2:** Results of the dean’s survey (*n* = 5)

	Total estimated hours teaching management of patients with obesity	Instruction Modalities Used	Domains of Obesity Addressed	Use of Non-Traditional Teaching Methods	Offer Dedicated Rotation in Clerkship focused on the Management of Patients with Obesity	Years in Which Management of Patients with Obesity is Taught
School 1	21.5	LGS, SGS, PBL, Clinical Simulation, Clinical Activity	Physical Activity, Nutrition, Behavioural Therapy, Pharmacotherapy, Surgery	Yes^a^	No	Years 1,2,3
School 2	12	LGS, SGS, Clinical Activity, Community-Based	Physical Activity, Nutrition, Pharmacotherapy, Surgery	None	No	Years 1,2
School 3	15	LGS, SGS, Clinical Simulation, Clinical Activity	Physical Activity, Nutrition, Behavioural Therapy, Pharmacotherapy, Surgery	None	No	Years 1,2,3,4
School 4	10	LGS, SGS, Clinical Activity, Community-Based	Nutrition, Surgery	None	No	Years 2,3,4
School 5	–	LGS, Clinical Activity	Physical Activity, Nutrition, Behavioural Therapy, Pharmacotherapy, Surgery	None	No	Years 1,2,3

*LGS* Large-group session, *SGS* Small-group session, *PBL* Problem-based learning

^a^In year 1, students participate in a hands-on cooking class, scavenger hunt-style grocery store tour, and self-directed in-store budgeting activity; facilitated by Registered Dietitians and Chefs. Goal: increase skills and confidence in managing eating habits, nutrition

## Discussion

### Summary of results

Our survey of English-speaking final-year medical students in Canada demonstrated limited knowledge and inadequate self-reported competence in managing patients with obesity. Our survey of UGME deans in Canada demonstrated an average of 14.6 curricular hours dedicated to teaching management of patients living with obesity across all years of medical school, with multiple instructional modalities used to deliver this content. Medical students were most knowledgeable on the etiology of obesity and goals of obesity treatment, and least knowledgeable on the physiology of obesity and long-term weight loss maintenance. We found no significant correlation between medical students’ knowledge scores and their self-reported competence in managing patients living with obesity. Together, these findings suggest a gap in UGME curricula regarding management of patients living with obesity.

### Explanation of findings

Our finding for the mean knowledge score of Canadian students (M = 10.5) is consistent with that of Martins and Norsett-Carr among Norwegian students (M = 10.8), and lower than the Norwegian experts’ score (M = 14.6) [26]. Canadian and Norwegian students demonstrated similar strengths and weaknesses, including greatest knowledge about the etiology of obesity and poorest knowledge about weight loss maintenance. It is encouraging that knowledge of the etiology of obesity was a relative strength among students, since this is associated with decreased weight bias and improved clinical interactions among practicing physicians [18, 27, 28]. Although the experts’ score may be used for comparison, the lack of validity evidence for a cut-off score for students limits our ability to draw conclusions about students’ overall clinical competence, although mean scores for performance on individual questions remains valuable for highlighting areas of weakness. The gaps in knowledge domains identified in Fig. 2 can be used as a guide by medical educators to develop future educational interventions aimed at the management of patients living with obesity. For example, only 4% of respondents correctly answered a knowledge question addressing the topic of diagnosing obesity in children. As such, future educational interventions may wish to consider addressing this topic. It is, however, promising that most students (80%) opted to receive personalized feedback, which suggests that survey research like ours on underrepresented topics may offer a good opportunity both to gather data, and to provide an educational intervention that is popular with medical students.

Our finding that graduating medical students report limited competence in managing patients living with obesity is novel; although consistent with those of Jay et al. who found similar results among staff physicians and residents in internal medicine, pediatrics, and psychiatry [13]. It is concerning that despite further medical training, staff and residents’ scores in Jay et al’s study were similar to those of medical students in our study. This finding reinforces the need for additional education on management of patients living with obesity at the UGME, post-graduate medical education, and continuing professional development levels. Future competency-based medical educational interventions should aim to address the areas of weakness identified in Table 1. For example, we would recommend including training in behavior change counseling as a strategy to improve perceived and objective competence levels of medical students in addressing topics of psychosocial comorbidities of obesity, goal setting, motivational interviewing, and counselling. In a 2014 systematic review, Sherson et al. described a misalignment between patients’ desires and physicians’ approach to weight management, highlighting the need for additional training in behavior change counseling [29].

We identified a discrepancy between students’ self-reported competence in assessing patients’ diet using tools like the FFQ and assessing diet for common unhealthy behaviors associated with obesity. This discrepancy highlights a lack of familiarity among medical students with standardized evidence-based tools available to assess a patient’s diet. Familiarity with such tools should become a formal part of competency-based education on the topic of obesity for Canadian medical students.

Our results from the UGME dean’s survey demonstrate that, in medical schools that responded, relatively few curricular hours are dedicated to the management of patients living with obesity, with variability in the topics covered. All schools discussed nutrition and surgery in their curriculum, but topics of physical activity, behavioral therapy and pharmacotherapy were less frequently addressed. By comparison, medical students spend more time learning about obesity-related comorbidities – for example, averaging 15.4 hours for diabetes alone [30] – which stands in contrast to the 14.5 hours spent on the chronic disease of obesity itself. In a similar study conducted across 141 medical schools in the United States, Butsch and colleagues reported that medical schools spend an average of only 10 hours on obesity education, during the entire 4-year undergraduate curriculum [25]. These findings are consistent with recent studies which demonstrate a persistently low prioritization of obesity-related education in contemporary medical school curricula despite ongoing calls for improvement [31–33].

### Future directions

A number of educational interventions are effective at improving medical students’ competence in managing patients living with obesity [19]. Such interventions should be incorporated and evaluated within the medical school curricula to address the competencies future physicians will require to meet the changing needs of their patients. Importantly, educational interventions should meet identified gaps. For example, the Association of American Medical Colleges in 2007 and the UK Royal College of Physicians in 2010 both described recommendations for obesity education reform at the UGME level [31, 34]. Similar initiatives should be adopted in Canada to meet the needs of Canadian medical students and patients. The recently published Clinical Practice Guideline for adults living with obesity [28] can serve as a guide for developing competencies (entrustable professional activities) within the competency-based UGME curriculum [35]. In addition, formal assessment of obesity-related competencies should be conducted throughout medical training and during formal licensing exams [20, 34].

In addition, future survey-based studies of educational interventions should consider including embedded personalized feedback as a way to support learning; and future research studies should explore whether there is an association between survey respondents’ performance and their propensity to request feedback on their responses.

### Limitations

First, participation in our study was voluntary and the response rate was 10.7%, which did not meet the threshold indicated by our power analysis (see Additional file 1), making our results subject to response bias and selection bias. Some medical schools declined to participate, and we only surveyed English-speaking schools, limiting the generalizability of our findings. The 36% response rate by UGME deans also limits the generalizability of our findings. Second, our study relied on students’ self-assessment of their own competencies, which is susceptible to self-report bias; however, objective knowledge scores were used to complement self-report data, increasing overall reliability. Third, fewer students completed the knowledge part of the questionnaire compared to the self-reported competence part, limiting the conclusions which can be drawn from within-subject analysis of knowledge and self-reported competence. Fourth, the dean’s survey relied upon respondents’ understanding of each domain of obesity management to accurately self-report the content of the obesity management curriculum, which we were unable to verify. Fifth, the absence of validity evidence for cut-off points for the student surveys limits our ability to make inferences about students’ overall clinical competence. Lastly, we were unable to match responses from the deans’ and the students’ surveys, due to confidentiality constraints, precluding us from drawing connections between program characteristics and students’ performance on the knowledge and competence questionnaires.

## Conclusions

Graduating Canadian medical students demonstrate limited knowledge and competencies in managing patients living with obesity. Current UGME curricula devote limited time to teaching such competencies. Further research should focus on defining, implementing, and assessing specific competencies focused on the management of patients living with obesity within the undergraduate competency-based medical curriculum. Instituting such changes will ensure that future physicians are better prepared to serve members of Canadian society living with obesity.

## Supplementary Information

**Additional file 1.** Protocol. Initial study protocol.

**Additional file 2.** CHERRIES. Checklist for reporting results of internet e-surveys.

**Additional file 3.** Medical Student Knowledge Questionnaire. 18-item questionnaire assessing students’ knowledge of obesity-management topics, adapted from Obesity Knowledge among Final-Year Medical Students in Norway, by C. Martins and A. Norsett-Carr (2017) [26].

**Additional file 4.** Medical Student Self-assessed Competency Questionnaire. 15-item questionnaire assessing students’ self-perceived competence in managing patients with obesity, adapted from Do Internists, Pediatricians, and Psychiatrists Feel Competent in Obesity Care?, by Jay et al. (2008) [13].

**Additional file 5.** Medical Student Knowledge Questionnaire Feedback Evidence-based explanations for correct and incorrect answer choices to the questions in the Medical Student Knowledge Questionnaire.

**Additional file 6.** UGME Dean’s Questionnaire. 7-item Questionnaire sent to undergraduate medical school deans of their delegates, assessing curricular teaching of material related to management of patients with obesity.

**Additional file 7.** Raw Data. Raw Data gathered and used to support the conclusions in this article. Data has been de-identified by redacting: 1. Submission IP addresses; 2. Location longitude and latitude; 3. Participant-submitted email addresses.

## Data Availability

The datasets supporting the conclusions of this article are included within the article (and its additional files; see “Additional_file_7”).

## Abbreviations

BMI: Body Mass Index
COVID-19: Coronavirus Disease 2019
UGME: Undergraduate Medical Education
CHERRIES: Checklist for Reporting Results of Internet E-Surveys
REB: Research Ethics Board
CAD: Canadian Dollars
M: Mean
SD: Standard Deviation
FFQ: Food Frequency Questionnaire
