# Supplementary material for: A Canadian survey of medical students and undergraduate deans on the management of patients living with obesity
Source: BMC Med Educ. 2022 Jul 21;22:562. doi: 10.1186/s12909-022-03636-9 (PMC9302212; doi:10.1186/s12909-022-03636-9)
Supplement: Supplementary file 1 — Additional file 1. Protocol. Initial study protocol. [file 12909_2022_3636_MOESM1_ESM.docx]

**Protocol**

**Purpose**

The purpose of our research study is to: (1) determine how management of patients with obesity is taught at the UGME level in Canada, and (2) determine the perceived level of competence of graduating medical students in managing patients with obesity.

**Hypothesis**

We hypothesize that management of patients with obesity makes up less that 5% of the educational curriculum in Canadian medical schools. We also hypothesize that graduating medical students in Canada feel unprepared to manage patients with obesity.

**Justification**

Obesity, defined as a body mass index (BMI) ≥ 30 kg/m^2^, is a rising public health concern in Canada [1]. Common obesity comorbidities such as hypertension, type II diabetes, and cancer, decrease quality of life and increase mortality [2, 3]. Despite the importance of obesity-management for patients, primary-care providers (PCPs) are poorly prepared to manage patients with obesity; only 32.1% of PCPs in Ontario (Canada) report having the necessary resources to effectively manage obese patients [4]. One study found that while most (85%) PCPs felt obligated to educate patients on the health risks of obesity, fewer than half (49%) felt competent in prescribing weight-loss measures as treatment [5]. PCPs feel ill-prepared to discuss a number of obesity-management options with their patients, including prescribing exercise [6], and referral for bariatric surgery [4, 7-10]. Negative attitudes of PCPs towards patients with obesity is a barrier to effective and timely treatment [8, 11, 12], whereas increase in knowledge about management of patients with obesity is associated with higher rates of referrals of patients for bariatric surgery [11]. Reflecting this sentiment, there a desire among PCPs for additional education in management of patients with obesity. A survey of primary care physicians in Ontario reported that 92.5% would like more education about bariatric surgery [4], and a national survey of US primary care physicians reported a desire by physicians to have more training in management of patients with obesity during their medical training [13].

Presently, there appears to be a gap in the education of undergraduate medical students on approach to and management of patients with obesity. The majority of graduating medical students regard weight-counselling for obese patients to be relevant to their intended practice [14]; however, most obesity-coded items that are tested on the US Medical Licencing Exam relate to common comorbidities of obesity, rather than holistic management of patients with obesity [15]. Improvement in students’ understanding of obesity can reduce the negative bias towards patients with obesity [16-18]. With over 20% of adults in Canada having class I obesity [19], there is an urgent need to identify if and how management of patients with obesity is being taught at an undergraduate medical education (UGME) level as our future physicians will need to be well versed in management of the chronic disease of obesity.

Despite the consensus in the literature that medical students should be taught approaches to the management of patients with obesity at the UGME level [10, 13, 15, 16, 18, 20-24], few studies have explored what is currently being taught at the UGME level, and none have explored the UGME in Canada.

**Objectives**

1. 1. To explore how management of patients with obesity is taught at the Undergraduate Medical Education (UGME) level in Canada by surveying Undergraduate Medical Deans about how this topic is addressed within their school’s curriculum;
2. 2. To determine the level of competence of graduating Canadian medical students in managing patients with obesity using a validated 18-item obesity-knowledge questionnaire;
3. 3. To determine the self-perceived level of competence of graduating Canadian medical students in managing patients with obesity using a 15-item questionnaire.

**Methods**

We will administer two national questionnaires to address our research questions. The first will be an online questionnaire distributed to the offices of the undergraduate deans at each of the 14 English-language medical schools in Canada. The questionnaire will explore how management of patients with obesity is currently taught in the UGME curriculum across several domains: number of hours spent on teaching obesity management; modalities used to teach this content (lecture, patient-interaction, group learning, independent learning, simulation-based learning, online modules, community-based learning); and the presence of any programs with a specific focus on the management of patients with obesity.

The second online questionnaire will be distributed to all graduating year medical students at the 14 English-language Canadian medical schools. This questionnaire will explore students’ actual and perceived competence in managing patients with obesity. Actual competence will be assessed by exploring students’ knowledge about lifestyle changes, medications, and bariatric surgery for management of obesity. We will assess perceived competence with questions grouped into three domains: Role Legitimacy, “is managing patients with obesity a part of my job?”; Role Competency, “do I have the knowledge and skills to effectively manage patients with obesity?”; and Resource Adequacy, “do I have the resources to effectively manage patients with obesity?”[25, 26]. Responses will be measured on a 5-point Likert scale from *strongly agree* to *strongly disagree*. A $200 lottery reward will be offered as an incentive for participation.

**Recruitment**

Graduating medical students are identified as belonging to a particular program. The 14 English-speaking medical schools in Canada is public knowledge; correspondence with each of the universities on their internal process for disseminating surveys to undergraduate medical students will inform who is eligible and how to advertise for recruitment into the current study. Individual medical schools will use their internal processes to disseminate the information about the survey and the link to the survey itself (e.g. through their class president, or Aesculapian Society, or internal portal, or local research lead).

Recruitment occurs between August 15^th^ 2019 and August 15^th^ 2020. Dissemination of surveys has occurred at some institutions already contacted. For some institutions, the process of disseminating the surveys and/or of applying for and receiving local ethics is still ongoing, partly due to delays from the COVID pandemic.

**Participants**

Participants fall into two categories: 1. Graduating medical students, and 2. Staff from Undergraduate Medical Education Offices of the Dean. For (1), Inclusion Criteria: Graduating medical students at English-speaking Canadian medical schools. Exclusion Criteria: Graduating medical students at exclusively French-speaking Canadian medical schools.

Inclusion Criteria justification: Graduating medical students are justified as participants in this research, as they are the only population capable of answering a research question regarding self- assessment of medical student preparedness for delivering care. Moreover, this study's findings will predominantly benefit its selected population by providing those medical students with an assessment of their own capabilities regarding treatment of patients with obesity, which will inform their skill development as future clinicians. Staff of the Undergraduate Medical Education Dean's offices are justified as being in a position to provide information on the curriculum, as indicated by the questionnaire (see documents).

Exclusion Criteria justification: Graduating medical students at exclusively French-speaking Canadian medical schools were excluded from the study, as the questionnaires being used were not validated in French and no appropriate bilingual validated questionnaires were found when designing the study.

**Sample Size Calculation**

The sample size for the UGME deans’ is 14 across Canada. The total sample size for class of 2020 graduating medical students in the 14 English-speaking Canadian medical schools is approximately 2,230 students. We anticipate a 15-30 percent response rate for medical students [27], yielding 335-670. For students, our anticipated response rate (based on prior survey data) of between 335 and 670 suggests that we will exceed the threshold of 328 responses necessary for statistical significance indicated by the sample size calculation below. Sample size calculation = ((z^2 x p (1 - p)) / e^2) / (1 + ((z^2 x p (1 - p)) / (e^N))) Where: N = population size (2,230), e = margin of error (0.05), z = z-score (at alpha = .05).

**Plan for Data Analysis**

Descriptive statistics (mean, standard deviation, median, range, 95% confidence interval) will be reported. Pearson Chi-squared tests will be used to compare categorical variables. One-way analysis of variance (ANOVA) will be used to compare continuous variables. Responses for ordinal outcomes on the Undergraduate Dean’s questionnaire and student questionnaire will be correlated using a Spearman rank-order correlation coefficient. Significance level will be set at α = .05 for all analyses. Statistical analysis will be completed using SPSS version 25.

**Significance**

Our study will identify how management of patients with obesity is currently being taught to medical students in Canada. We will also assess whether graduating medical students feel competent in managing patients with obesity at the end of their medical school training. The results of our study can inform a change in the UGME curriculum to provide future physicians with knowledge and skills to manage patients with obesity in their future practice. This in turn is expected to improve access to care for patients with obesity.

**References**

1. Twells, L.K., et al., *Current and predicted prevalence of obesity in Canada: a trend analysis.* CMAJ Open, 2014. **2**(1): p. E18-26.

2. Guh, D.P., et al., *The incidence of co-morbidities related to obesity and overweight: a systematic review and meta-analysis.* BMC Public Health, 2009. **9**: p. 88.

3. Luo, W., et al., *The burden of adult obesity in Canada.* Chronic Dis Can, 2007. **27**(4): p. 135-44.

4. Auspitz, M., et al., *Knowledge and Perception of Bariatric Surgery Among Primary Care Physicians: a Survey of Family Doctors in Ontario.* Obes Surg, 2016. **26**(9): p. 2022-2028.

5. Foster, G.D., et al., *Primary care physicians' attitudes about obesity and its treatment.* Obes Res, 2003. **11**(10): p. 1168-77.

6. O'Brien, M.W., et al., *Health care provider confidence and exercise prescription practices of Exercise is Medicine Canada workshop attendees.* Applied Physiology Nutrition and Metabolism, 2017. **42**(4): p. 384-390.

7. Avidor, Y., et al., *Primary care and subspecialty management of morbid obesity: referral patterns for bariatric surgery.* Surg Obes Relat Dis, 2007. **3**(3): p. 392-407.

8. Balduf, L.M. and T.M. Farrell, *Attitudes, beliefs, and referral patterns of PCPs to bariatric surgeons.* J Surg Res, 2008. **144**(1): p. 49-58.

9. Funk, L.M., et al., *Patient and Referring Practitioner Characteristics Associated With the Likelihood of Undergoing Bariatric Surgery: A Systematic Review.* JAMA Surg, 2015. **150**(10): p. 999-1005.

10. Tork, S., et al., *Factors Influencing Primary Care Physicians' Referral for Bariatric Surgery.* JSLS, 2015. **19**(3).

11. Ferrante, J.M., et al., *Family physicians' practices and attitudes regarding care of extremely obese patients.* Obesity (Silver Spring), 2009. **17**(9): p. 1710-6.

12. Phelan, S.M. and T.I. Rajjo, *Trainees' experiences and attitudes and the delivery of patient-centred care for obesity.* Med Educ, 2017. **51**(8): p. 775-777.

13. Bleich, S.N., et al., *National survey of US primary care physicians' perspectives about causes of obesity and solutions to improve care.* Bmj Open, 2012. **2**(6).

14. Rose, A.E., E. Frank, and J.S. Carrera, *Factors affecting weight counseling attitudes and behaviors among U.S. medical students.* Acad Med, 2011. **86**(11): p. 1463-72.

15. Kushner, R.F., et al., *Obesity Coverage on Medical Licensing Examinations in the United States. What Is Being Tested?* Teach Learn Med, 2017. **29**(2): p. 123-128.

16. Gayer, G.G., J. Weiss, and M. Clearfield, *Fundamentals for an Osteopathic Obesity Designed Study: The Effects of Education on Osteopathic Medical Students' Attitudes Regarding Obesity.* J Am Osteopath Assoc, 2017. **117**(8): p. 495-502.

17. Poustchi, Y., et al., *Brief intervention effective in reducing weight bias in medical students.* Fam Med, 2013. **45**(5): p. 345-8.

18. Roberts, D.H., et al., *Teaching medical students about obesity: a pilot program to address an unmet need through longitudinal relationships with bariatric surgery patients.* Surg Innov, 2011. **18**(2): p. 176-83.

19. Statistics Canada, *Overweight and obese adults (self-reported), 2014.* Publications, 2014. **82-625-X**. https://www150.statcan.gc.ca/n1/pub/82-625-x/2015001/article/14185-eng.htm (accessed Feb 14, 2019).

20. Bessesen, D.H., *It Is Time to Put Obesity Management into the Medical Curriculum.* Obesity Management, 2006. **2**(5): p. 169-172.

21. Kushner, R.F., et al., *An obesity educational intervention for medical students addressing weight bias and communication skills using standardized patients.* BMC Med Educ, 2014. **14**: p. 53.

22. Martins, C. and A. Norsett-Carr, *Obesity Knowledge among Final-Year Medical Students in Norway.* Obes Facts, 2017. **10**(6): p. 545-558.

23. Tsai, A.G., et al., *Evidence of a gap in understanding obesity among physicians.* Obes Sci Pract, 2018. **4**(1): p. 46-51.

24. Vitolins, M.Z., et al., *Obesity educational interventions in U.S. medical schools: a systematic review and identified gaps.* Teach Learn Med, 2012. **24**(3): p. 267-72.

25. Leedham-Green, K.E., R. Pound, and A. Wylie, *Enabling tomorrow's doctors to address obesity in a GP consultation: an action research project.* Educ Prim Care, 2016. **27**(6): p. 455-461.

26. Nolan, C., et al., *Practice nurses and obesity: professional and practice-based factors affecting role adequacy and role legitimacy.* Prim Health Care Res Dev, 2012. **13**(4): p. 353-63.

27. Gerbase, M.W., et al., *How Many Responses Do We Need? Using Generalizability Analysis to Estimate Minimum Necessary Response Rates for Online Student Evaluations.* Teach Learn Med, 2015. **27**(4): p. 395-403.
