# Supplementary material for: A Canadian survey of medical students and undergraduate deans on the management of patients living with obesity
Source: BMC Med Educ. 2022 Jul 21;22:562. doi: 10.1186/s12909-022-03636-9 (PMC9302212; doi:10.1186/s12909-022-03636-9)
Supplement: Supplementary file 3 — Additional file 3. Medical Student Knowledge Questionnaire. 18-item questionnaire assessing students’ knowledge of obesity-management topics, adapted from Obesity Knowledge among Final-Year Medical Students in Norway, by C. Martins and A. Norsett-Carr (2017) [26]. [file 12909_2022_3636_MOESM3_ESM.docx]

**Medical Student Knowledge Questionnaire**

**Table 1.** Questionnaire – please choose only one answer for each one of the questions below

*Physiology*
1. Which of the following alternatives best characterizes people with obesity (BMI ≥ 30 kg/m^2^), compared to normal-weight individuals?

A. A lower resting metabolic rate (RMR)
B. Lower total energy expenditure (TEE) during physical activity given similar intensity and duration

C. A higher TEE
D. A decrease in carbohydrate metabolism

2. Which of the following alternatives is correct in terms of TEE and RMR?

A. Degree of fat-free mass (FFM) is crucial for RMR

B. RMR is not affected by gender, age or BMI (kg/m^2^)
C. RMR accounts for approximately 50% of TEE in inactive individuals with obesity
D. Exercise-induced energy expenditure accounts for approximately 50% of TEE in an inactive individual with obesity

*Etiology*

3. Which of the following alternatives is considered to be the main reasons for an increase in overweight and obesity?

A. Lack of self-control
B. Genetics
C. Genetic predisposition in addition to inactivity and overabundance of food

D. Increasing use of medications that can lead to weight gain

E. Endocrine causes

4. Weight gain (WG) after a period of weight loss (WL), is one of the most profound challenges in obesity management. Which of the following alternatives represents the most likely contributor?

A. Reduction in motivation and lack of compliance
B. Reduction in RMR and a decrease in energy expenditure related to PA
C. Increase in hunger sensation and a decrease in satiety due to physiological adaptations to appetite control systems D. Combination of A+B+C

*Diagnosis*

5. Which diagnostic criterion regarding obesity represents the current standard

A. BMI (kg/m^2^)
B. Presence of comorbidities
C. Body composition (fat-free mass vs. fat mass)

D. Amount of visceral adipose tissue (VAT)

6. When diagnosing obesity in children, which of the following tools is considered to be the best one to use?

A. BMI curve
B. Waist-to-hip ratio

C. Iso-BMI curve
D. Percentiles

7. Which of the patients would you most likely prioritize in terms of treatment for obesity?

A. Female 38 years old, BMI 50 kg/m^2^, mild hypertension, knee and lower back pain
B. Male 34 years old, BMI 35 kg/m^2^, diabetes type II, obstructive sleep apnea (OSAS)
C. Female 48 years old, BMI 32 kg/m^2^, physically active, minor joint discomforts

D. Male 36 years old, BMI 45 kg/m^2^, impaired fasting glucose, mild depression

*Goals for Obesity Treatment*

8. Which alternative in terms of reduction in body weight is considered to give significant improvements in health?

A. 10–15 kg WL
B. 5–10% WL from baseline weight
C. A reduction in BMI category (e.g. from WHO class III to WHO class II)

D. A reduction in waist circumference (cm) by 10%

*Conservative Treatment of Obesity*

9. What is considered to be the most optimal form for exercise in treating obesity?

A. 4 × 4 high-intensity interval training (HIIT)
B. Combined endurance and resistance exercise
C. Resistance exercise

D. Exercising in the moderate intensity zone/fat burning zone

10. What is considered to be the most optimal strategy for lifestyle treatment of obesity?

A. Changing dietary habits
B. Combination of diet and exercise
C. Increasing physical activity levels (PALs)

D. Cognitive behavioral therapy (CBT)
E. Combination of diet, exercise and CBT

11. When considering long-term weight reduction, which diet is believed to be the most effective one?

A. Low carbohydrate – high fat (LCHF)
B. Low fat
C. Mediterranean diet

D. Any diet can give the same weight reduction given equal negative energy balance and long-term compliance

12. Which of the following alternatives is considered to be the most appropriate recommendation when looking at conservative treatment of obesity?

A. A negative energy deficit of approximately 600 kcal/day
B. <20% of the energy in the diet comes from fat as a macronutrient

C. A weight loss of >1.0 kg/week
D. A diet very low in energy (<800 kcal/day)

*Surgery (Non-Conservative Treatment of Obesity)*
13. Which of the following alternatives is the most correct one when looking at long-term outcomes of surgical treatment of obesity (gastric bypass, GBP)?

A. GBP improves the metabolic risk profile, but not primarily cardiovascular risk
B. Approximately 15% of patients experience suboptimal weight loss or significant weight regain
C. GBP does not produce a more significant WL after two years when compared to lifestyle treatment of obesity

D. Approximately 95% of patients who undergo GBP respond well when looking at WL

14. Which of the following alternatives represents the most common complication experienced after GBP?

A. Hypertension
B. Dyslipidemia
C. Low levels of vitamin B12, vitamin D, calcium, and iron

D. Osteoporosis

*Consequences of Obesity*

15. Which of the following alternatives are least associated with obesity?

A. Diabetes Mellitus Type II (DM2)

B. Osteoporosis
C. Male infertility
D. Non-alcoholic fatty liver disease (NAFLD)

*Weight Loss Maintenance – Long-Term Perspective*

16. Which level of physical activity is recommended for individuals with obesity in order to maintain weight loss?

A. 30 min/day moderate intensity
B. Short 10 min bouts with high intensity 3 times/week
C. 45–60 min/day moderate intensity

D. 30 min HIIT 3 times/week

17. On average, which percentage do individuals who have lost weight through lifestyle changes are able to maintain a clinically significant WL for at least 1 year?

A. 20%

B. <10%

C. 30%

D. >40%

18. Which of the following alternatives is most associated with long-term WL maintenance?

A. A diet high in carbohydrates (≥55% of total energy intake)
B. Exercising at high intensity > 3 times/week
C. Eating breakfast > 5 days/week

D. Self-weighing ≥ 1 times/month

*Note*. Reprinted from *Obesity Knowledge among Final-Year Medical Students in Norway*, by C. Martins and A. Norsett-Carr.
