# Supplementary material for: A Canadian survey of medical students and undergraduate deans on the management of patients living with obesity
Source: BMC Med Educ. 2022 Jul 21;22:562. doi: 10.1186/s12909-022-03636-9 (PMC9302212; doi:10.1186/s12909-022-03636-9)
Supplement: Supplementary file 4 — Additional file 4. Medical Student Self-assessed Competency Questionnaire. 15-item questionnaire assessing students’ self-perceived competence in managing patients with obesity, adapted from Do Internists, Pediatricians, and Psychiatrists Feel Competent in Obesity Care?, by Jay et al. (2008) [13]. [file 12909_2022_3636_MOESM4_ESM.docx]

**Medical Student Self-Assessed Competency Questionnaire**

For each question, indicate your level of confidence in being able to perform the task, according to the following scale:

| 1 | 2 | 3 | 4 |
| --- | --- | --- | --- |
|  |  |  |  |
| *Know very little about and not able to perform* | *Know something about and somewhat able to perform* | *Able to perform well* | *Able to teach others how to perform* |

***I can…***

1. Use 24-hour recall, food record, or food frequency to obtain diet history

| 1 | 2 | 3 | 4 |
| --- | --- | --- | --- |
| ☐ | ☐ | ☐ | ☐ |

1. Determine body mass index (BMI) from weight and height measurements

| 1 | 2 | 3 | 4 |
| --- | --- | --- | --- |
| ☐ | ☐ | ☐ | ☐ |

1. Assess diet for common unhealthy behaviours associated with obesity (e.g. sweetened beverages, nutritional quality of snacks, frequent meals from fast food restaurants, etc.)

| 1 | 2 | 3 | 4 |
| --- | --- | --- | --- |
| ☐ | ☐ | ☐ | ☐ |

1. Ascertain each patient’s readiness and ability to work on weight loss according to health beliefs and stage of change

| 1 | 2 | 3 | 4 |
| --- | --- | --- | --- |
| ☐ | ☐ | ☐ | ☐ |
|  |  |  |  |

1. Recognize and screen for common psychosocial problems in obese patients including depression, emotional eating, and binge eating

| 1 | 2 | 3 | 4 |
| --- | --- | --- | --- |
| ☐ | ☐ | ☐ | ☐ |

Take a targeted history and conduct a physical examination to identify common co-morbidities (e.g. arthritis, diabetes, PCOS…)

| 1 | 2 | 3 | 4 |
| --- | --- | --- | --- |
| ☐ | ☐ | ☐ | ☐ |

1. Discuss the effect of obesity on present and future health and personalize risk to each patient

| 1 | 2 | 3 | 4 |
| --- | --- | --- | --- |
| ☐ | ☐ | ☐ | ☐ |

1. Respond to a patient’s questions regarding treatment options including behaviour change, medications, and surgery

| 1 | 2 | 3 | 4 |
| --- | --- | --- | --- |
| ☐ | ☐ | ☐ | ☐ |

1. Assess current level of physical activity and provide guidance for setting physical activity goals for optimal health

| 1 | 2 | 3 | 4 |
| --- | --- | --- | --- |
| ☐ | ☐ | ☐ | ☐ |

1. Assist patient in setting realistic goals for weight loss based on making permanent lifestyle changes

| 1 | 2 | 3 | 4 |
| --- | --- | --- | --- |
| ☐ | ☐ | ☐ | ☐ |

1. Prescribe plan for exercise / physical activity

| 1 | 2 | 3 | 4 |
| --- | --- | --- | --- |
| ☐ | ☐ | ☐ | ☐ |

1. Use motivational interviewing to change behaviour

| 1 | 2 | 3 | 4 |
| --- | --- | --- | --- |
| ☐ | ☐ | ☐ | ☐ |

1. Provide brief counseling intervention to help patient lose weight

| 1 | 2 | 3 | 4 |
| --- | --- | --- | --- |
| ☐ | ☐ | ☐ | ☐ |

1. Recognize and refer patients with eating disorders

| 1 | 2 | 3 | 4 |
| --- | --- | --- | --- |
| ☐ | ☐ | ☐ | ☐ |

1. Collaborate with registered dieticians and refer to community nutrition resources when appropriate

| 1 | 2 | 3 | 4 |
| --- | --- | --- | --- |
| ☐ | ☐ | ☐ | ☐ |

*Note*. Adapted from *Do Internists, Pediatricians, and Psychiatrists Feel Competent in Obesity Care?*, by Jay *et al*. (2008).
