# Supplementary material for: A Canadian survey of medical students and undergraduate deans on the management of patients living with obesity
Source: BMC Med Educ. 2022 Jul 21;22:562. doi: 10.1186/s12909-022-03636-9 (PMC9302212; doi:10.1186/s12909-022-03636-9)
Supplement: Supplementary file 5 — Additional file 5. Medical Student Knowledge Questionnaire Feedback Evidence-based explanations for correct and incorrect answer choices to the questions in the Medical Student Knowledge Questionnaire. [file 12909_2022_3636_MOESM5_ESM.docx]

**Medical Student Knowledge Questionnaire Feedback**

*The following text provides explanations for the correct and incorrect answers to the 18-knowledge based questions in* **Additional_File_3 – Medical Student Knowledge Questionnaire***. These explanations were embedded into the Qualtrics survey itself, the platform we used to disseminate our survey.*

*These explanations were offered to participants after they finished entering all responses to the survey. They were optional, that is, upon being given the option to read these explanations, participants may have chosen to do so, or they may have chosen to exit the survey. The intent behind these explanations was to provide interested students the opportunity to learn more about obesity management in clinical practice, and to provide students with personalized feedback on their performance of the questionnaire they had just completed.*

1. Which of the following best characterizes people with obesity (BMI ≥ 30 kg/m^2^) compared to normal-weight individuals?

A. A lower resting metabolic rate (RMR)

B. Lower total energy expenditure (TEE) during physical activity given similar intensity and duration

**C. A higher total energy expenditure (TEE)**

D. A decrease in carbohydrate metabolism

Option A is incorrect. Although it was historically thought that obesity may be driven by lower RMR resulting in an energy surplus and thus weight gain, more recent studies do not support this hypothesis. Numerous recent studies have found that obesity is actually associated with a higher RMR, the rationale being that increased fat body mass produces a compensatory increase in Fat-Free Mass (FFM) tissue, such as brain, skeletal muscle, and bone, which is highly metabolically demanding [1-3]. In short, a greater body mass requires more calories in order to function at baseline, so people with obesity typically have a higher RMR than non-obese people. Carneiro et al.’s (2016) review article offers a good summary of the recent literature [1, 4].

Option B is incorrect, for similar reasons as Option A - during physical activity of similar intensity and duration, a person with obesity requires more energy than a person without obesity, as their skeletal muscles is put under greater metabolic demands to move the increased FFM tissue and Fat Mass (FM) tissue. Interestingly, this is an absolute difference only; when adjusting for increased FFM and FM, obese and non-obese individuals have similar relative TEE during physical activity. In summary, having obesity is associated with higher absolute TEE during physical activity [4, 5].

**Option C, "A higher total energy expenditure" is the correct answer, since it is the only answer that universally characterizes individuals with obesity. The increased metabolic demands of greater FFM and FM in individuals with obesity results in a higher total energy expenditure [1].**

Option D is incorrect – the opposite is true. Obesity is associated with increased carbohydrate metabolism at rest, especially in the case of high-carbohydrate diets. Increased carbohydrate metabolism is driven by increasing insulin resistance characteristic of individuals with obesity [6].

2. Which of the following is correct in terms of total energy expenditure (TEE) and resting metabolic rate (RMR)?

**A. Degree of fat-free mass (FFM) is crucial for RMR**

B. RMR is not affected by gender, age or BMI

C. RMR accounts for approximately 50% of TEE in inactive individuals with obesity

D. Exercise-induced energy expenditure accounts for approximately 50% of TEE in inactive individuals with obesity

**Option A is correct. Degree of Fat Free Mass (FFM) is crucial for determining Resting Metabolic Rate (RMR), since the FFM compartment is composed of metabolically demanding tissue such as brain, skeletal muscle, and bone [3].**

Option B is incorrect. Resting Metabolic Rate is affected by gender, age, and BMI. Male children and adolescents have higher RMR compared to females, although RMR does not differ between male and female adults [7]; RMR decreases with age, almost linearly [8]; and a higher Body Mass Index (BMI) is associated with higher RMR [1].

Option C is incorrect. Resting Metabolic Rate is well-established as the largest single factor of Total Energy Expenditure and is estimated at between 50-75%. Normal-weight individuals engaging in daily physical activity have an RMR closer to 50%, while obese individuals have an RMR closer to 75% [1, 9].

Option D is incorrect. Given that RMR accounts for approximately 70% of TEE in obese individuals, no other factor can account for more than 30% of TEE. In addition, the population in the question is ‘inactive’ individuals with obesity, which implies that Exercise-induced Energy Expenditure (EEE) would be low. For an individual with obesity, EEE is likely to constitute around 27% of TEE [10].

3. Which of the following is considered to be the main reason for an increase in obesity?

A. Lack of self-control

B. Genetics

**C. Genetic predisposition in addition to inactivity and overabundance of food**

D. Increasing use of medications that can lead to weight gain

E. Endocrine causes

Option A is incorrect. Although there is research supporting the idea that self-control is an individual difference variable which may influence eating behaviours [11, 12], “self-control” is difficult to quantify, and is not considered to be one of the larger contributing factors. Given the complex physiological processes that govern weight loss and weight gain, there is scientific consensus that ‘lack of self-control’ is unlikely to be the *main* reason for increasing rates of obesity [13].

Option B is incorrect. Although genetics play a significant role in the development obesity, genetics alone cannot be considered the ‘main’ reason, when compared to Option C, “genetic predisposition in addition to inactivity and overabundance of food” [14].

**Option C is correct. Genetic predisposition plays a significant role in the development of obesity [14], but the fundamental cause of obesity is greater energy intake than energy expenditure over an extended period of time – in other words, a net calorie excess [13]. Genetic predisposition for obesity can promote excess weight via hormonal, behavioural, biological, and other mechanisms; inactivity results in lower Total Energy Expenditure, and an overabundance of food results in higher total calories, both of which increase the likelihood of a net calorie excess and subsequent weight gain. Overabundance of food and increasing inactivity due to evolutionarily unprecedented population-level changes in dietary habits, food sources, and income, are thought to play a significant role in the increasing rates of obesity worldwide [13].**

Option D is incorrect. Although obesogenic medications can play a role in weight gain, they are not considered to be the main factor in increasing rates of obesity [15]. Obesogenic medications exercise their effects via cellular pathways that influence behavioural or metabolic outcomes. Regardless, the root cause of obesity – net calorie excess – cannot be directly caused by an obesogenic medication.

Option E is incorrect. Although the Endocrine system plays a major role in the homeostatic regulation of energy intake and expenditure, abnormalities in the functioning of the Endocrine system implied by “Endocrine causes” (such as hypogonadotropic hypogonadism) are relatively rare, typically produce severe obesity, and constitute only a small fraction of total cases of obesity [13].

4. Weight gain (WG) after a period of weight loss (WL), is one of the most profound challenges in obesity management. Which of the following alternatives represents the most likely contributor?

A. Reduction in motivation and lack of compliance

B. Reduction in resting metabolic rate (RMR) and a decrease in energy expenditure related to physical activity

C. Increase in hunger sensation and a decrease in satiety due to physiological adaptations to appetite control systems

**D. All of the above**

Option A, alone, is incorrect. Reduction in motivation and lack of compliance to a weight loss regimen following a period of weight loss does promote weight re-gain [12, 16], but this behavioural factor is only one contributor to weight re-gain.

Option B, alone, is incorrect. Although there is a reduction in resting metabolic rate and a decrease in energy expenditure during physical activity following weight loss [2, 17], this shift in energy homeostasis is only one factor contributing to weight re-gain.

Option C, alone, is incorrect. As in Options A and B, the statement is true. Following a period of weight-loss, a combination of hormonal, metabolic, and neural inputs act on CNS circuits in the brain, predominantly the Hypothalamus, to increase appetite and decrease satiety [18]. Examples of these regulators include hormones such as leptin, cortisol, and insulin, metabolites such as glucose, and vagal input from gut distension [13]. Nonetheless, these physiological adaptations to appetite control systems are only one factor contributing to weight re-gain.

**Option D is correct. Behavioural (Option A), Homeostatic (Option B), and Physiological (Option C), factors all contribute to weight re-gain following a period of weight loss.**

5. Which diagnostic criterion regarding obesity represents the current standard?

**A. Body Mass Index (BMI)**

B. Presence of comorbidities

C. Body composition (fat-free mass vs. fat mass)

D. Amount of visceral adipose tissue (VAT)

**Option A is correct. BMI is the most widely-used diagnostic criterion for obesity and represents the current standard. BMI is calculated by weight/height^2^ (in kg/m^2^). The term “overweight” is commonly used to describe individuals with a BMI between 25-29.9. A BMI of 30 is considered the threshold for “obesity” in both men and women [13].**

Option B is incorrect. Presence of comorbidities is not considered in the diagnostic criterion for obesity, although some studies find that the presence of comorbidities increases the probability of diagnosis and documentation of obesity in clinic and inpatient settings [19, 20].

Option C is incorrect. Fat Free Mass (FFM) and Fat Mass (FM) are predominantly used in determining Total Energy Expenditure (TEE), and for calculations involving Resting Metabolic Rate (RMR). Although calculation of FFM and FM can be used as a metric for obesity, it is not currently used as the diagnostic standard.

Option D is incorrect. Visceral Adipose Tissue (VAT) is one of the two compartments of total body fat (the other being Subcutaneous Adipose Tissue; SAT). VAT is a hormonally active tissue and is predominantly involved in energy storage and regulation. While the inappropriate accumulation of VAT is defined as Central or Abdominal obesity, measuring VAT alone does not provide a broad enough diagnostic criterion for obesity [21].

6. When diagnosis obesity in children, which of the following tools is considered to be the best one to use?

A. BMI curve

B. Waist-to-hip ratio

**C. Iso-BMI curve**

D. Percentiles

Option A is incorrect. A BMI curve is used to diagnose obesity in adults [22]. The age-and-sex-adjusted Body Mass Index (Iso-BMI) is a more precise tool than BMI for diagnosing obesity in children.

Option B is incorrect. Waist-to-hip ratio (WHR) is the ratio of the circumference of the waist to the circumference of the hips. WHR is one of three measures of central obesity, the others being waist circumference and waist-to-height ratio [23]. In adolescents, WHR is more weakly correlated with percentage body fat than is BMI, making WHR a less useful tool than BMI for diagnosing obesity in adolescents [24], although WHR plays an important role in diagnosing adult obesity [25]. Some studies support WHR as being a better predictor of cardiovascular events than BMI [26], however there is no consensus in the literature that among the 3 measures of central obesity and BMI, any one is the best predictor of future cardiovascular events [23].

**Option C is correct. Iso-BMI is an age-and-sex-adjusted Body Mass Index used for the pediatric population aged 2-18 years [27]. Cole and colleagues [28] first established threshold values for pediatric obesity in 2000, as part of the International Obesity Task Force (IOTF). The IOTF values have since been accepted as the best tool for diagnosing obesity in children, and the term Iso-BMI curve has become used to refer to these pediatric tables.**

Option D is incorrect. “Percentiles” implies threshold values on some continuous measure, such a BMI percentiles. BMI percentiles are uniformly used to diagnose obesity in children, although the variety of different reference data sets used worldwide gives rise to slightly differing definitions of “overweight” and “obesity” [29]. Moreover, studies have shown that the use of percentiles can underestimate the progression of weight gain and lead to underdiagnosis of obesity in children [30, 31]. Thus, there are numerous drawbacks to using percentiles as the foremost diagnostic tool for childhood obesity.

7. Which of the following patients would you most likely prioritize in terms of treatment for obesity?

A. Female 38 years old, BMI 50 kg/m2, mild hypertension, knee and lower back pain

**B. Male 34 years old, BMI 35 kg/m2, diabetes type II, obstructive sleep apnea (OSAS)**

C. Female 48 years old, BMI 32 kg/m2, physically active, minor joint discomforts

D. Male 36 years old, BMI 45 kg/m2, impaired fasting glucose, mild depression

Option A is incorrect. Answering this question requires knowledge of the current approach to prioritizing treatment for obesity. Until 2009, Body Mass Index (BMI) was predominantly used by clinicians to inform which patients received which treatments. However, criticism of BMI as overly arbitrary led Sharma and Kushner (2009) to propose the now-standard Edmonton Obesity Staging System (EOSS) [32]. The EOSS is a clinical staging system (similar to the well-known ‘tumor, node, metastasis’ [TNM] system used in oncology) which classifies the severity of obesity based on the presence of comorbidities, functional limitations, and risk factors [32]. By using clinical assessment rather than anthropomorphic measures to guide patient management, the EOSS better predicts mortality risk in patients with obesity [33, 34], and better guides selection of patients for bariatric surgery [35, 36]. Patient A has an EOSS score of 1 (presence of obesity-related subclinical risk factors), putting them lower priority than Patient B [32]. Management for EOSS score of 1 is *“Investigation for other (non-weight related) contributors to risk factors. More intense lifestyle interventions, including diet and exercise to prevent further weight gain. Monitoring of risk factors and health status.”* ([32] Table 4 pg 293).

**Option B is correct. Patient B has an EOSS score of 2 (presence of obesity-related chronic diseases), making them the highest priority of all the patients listed [32]. In addition, for a given BMI, males generally have a greater number of obesity-related comorbidities than females; consequently, a male patient with BMI 35, Type II Diabetes (T2DM), and OSAS, is at a higher risk of developing macrovascular and microvascular complications of T2DM, as well as pulmonary hypertension, and hypertension with OSAS [37]. In summary, a female with a higher BMI and mild obesity-related comorbidities (EOSS 1) should not take priority over a male with lower BMI and a greater number of obesity-related comorbidities (EOSS 2). Management for EOSS score of 2 is *“Initiation of obesity treatments including consideration of all behavioural, pharmacological and surgical treatment options. Close monitoring and management of comorbidities as indicated.”* ([32] Table 4 pg 293).**

Option C is incorrect. Patient C has an EOSS score of 0 (no apparent obesity-related risk factors), putting them lower priority than Patient B [32]. Management for EOSS score of 0 is *“Identification of factors contributing to increased body weight. Counselling to prevent further weight gain through lifestyle measures including healthy eating and increased physical activity.”* ([32] Table 4 pg 293).

Option D is incorrect. Patient D has an EOSS score of 1 (presence of obesity-related subclinical risk factors), putting them lower priority than Patient B [32].

8. Which alternative in terms of reduction in body weight is considered to give significant improvements in health?

A. 10-15 kg weight loss

**B. 5-10% weight loss from baseline weight**

C. A reduction in BMI category (e.g. from WHO class III to WHO class II)

D. A reduction in waist circumference (cm) by 10%

Option A is incorrect. Since individuals classified as “overweight” or “obese” may have differing weights, a uniform 10-15kg weight loss would constitute a different percentage of weight lost from baseline for different individuals. Significant improvements in health emerge across individuals of varying weight when a similar percent of weight is lost from baseline weight, not when an absolute amount of weight is lost [38].

**Option B is correct. A 5-10% weight loss from baseline weight is considered to be clinically significant weight loss. Benefits of 5-10% weight loss include improving lipid levels, improving insulin-stimulated glucose metabolism, improving blood pressure control, and reducing the risk of cardiovascular events [38-40].**

Option C is incorrect. A reduction in BMI category does not necessarily mean a significant amount of weight has been lost. There are three World Health Organization (WHO) classes of obesity, each defined by a BMI category: class I (BMI 30 – 34.9); class II (BMI 35 – 39.9); and class III (BMI > 40). If an individual’s BMI is near the threshold between two BMI categories, a small change in weight may change their BMI category, but may not be enough to give significant improvements in health [41].

Option D is incorrect. Although the all-cause mortality relative risk (RR) for a 10% increase in waist circumference among men is 1.36, and among women is 1.30 (and for a 5-unit increase in BMI it is 1.18), a 5-10% weight loss from baseline weight is considered to give more significant improvements in health than a 10% reduction in waist circumference [42, 43].

9. What is considered to be the optimal form of exercise for treating obesity?

A. 4 x 4 high-intensity interval training (HIIT)

**B. Combined endurance and resistance exercise**

C. Resistance exercise

D. Exercising in the moderate intensity zone / fat burning zone

Option A is incorrect. Although High-intensity Interval Training (HIIT) is a time-effective method for improving overall cardiovascular health, initial effects of HIIT are changes in body composition (reduced body fat percentage) but not changes in overall body weight [44]. There is conflicting evidence on whether HIIT is more effective than endurance training at increasing aerobic and anaerobic capacity, however HIIT is consistently reported as less enjoyable than endurance training [45, 46]. In addition, HIIT may not be accessible for patients with severe obesity.

**Option B is correct. Combined endurance and resistance exercise is superior to resistance exercise alone. The combination of exercise regimes has been found to improve body composition, reduce inflammation, and improve markers of metabolism [47], while reducing risk factors associated with diabetes and cardiovascular events [48].**

Option C is incorrect. Combined endurance and resistance exercise, Option B, is superior to resistance exercise alone [47-49].

Option D is incorrect. Moderate Intensity Training has been found to be less efficient than HIIT at achieving similar outcomes [44].

10. What is considered to be the optimal strategy for lifestyle treatment of obesity?

A. Changing dietary habits

B. Combination of diet and exercise

C. Increasing physical activity levels (PALs)

D. Cognitive behavioural therapy (CBT)

**E. Combination of diet, exercise and CBT**

Option A is incorrect. Changing dietary habits is an important component of lifestyle treatment of obesity, since the root cause of obesity is a net calorie excess and adjusting dietary habits can reduce daily total energy intake [13]. However, adherence to dietary changes alone is usually unsuccessful (Dansinger et al. [2005] report dietary adherence around 25% after 1 year [50]), and prescribing dietary changes has poorer outcomes in the absence of additional weight-management strategies. Combining dietary changes with exercise is more effective at producing weight loss than diet alone [51], while incorporating behavioural strategies in addition to diet and exercise is the most effective approach for producing and maintaining weight loss [52].

Option B is incorrect. Although a combination of diet and exercise may be successful at producing weight loss [51], weight loss is more frequently maintained when diet and exercise are combined with behavioural strategies for treatment of obesity [52].

Option C is incorrect. Although increasing physical activity levels (PALs) increases total energy expenditure, attenuating the daily calorie excess that is the root cause of obesity, merely increasing PALs alone is less successful at driving weight loss that is PALs combined with dietary and behavioural strategies [53]. In part, this is due to physiological changes that occur when increasing PALs that increase appetite and decrease satiety, making it more likely that more calories will be consumed, perpetuating the daily calorie excess [54].

Option D is incorrect. While cognitive behavioural therapy (CBT) is a valuable component of lifestyle treatment of obesity, addressing the root cause of obesity (net calorie excess) requires change of dietary and physical activity levels [52]. CBT is one of several methods of behaviour therapy for treating obesity, whose common goal is to help obese individuals to modify eating habits, physical activity levels, and thought processes, that contribute to excess weight. Key components of behavioural therapy include specific goal setting, and self-monitoring [52].

**Option E is correct. The optimal strategy for lifestyle treatment of obesity is a comprehensive program of lifestyle modification, also known as behavioural weight control. Lifestyle modification has three components: diet, exercise, and behaviour therapy [52].**

11. When considering long-term weight reduction, which diet is believed to be the most effective?

A. Low carbohydrate - high fat (LCHF)

B. Low fat

C. Mediterranean diet

**D. Any diet can give the same weight reduction given equal negative energy balance and long-term compliance**

Option A is incorrect. Low carbohydrate – high fat (LCHF) diets, such as the Atkins diet, are typically low in carbohydrate (less than 20 grams per day), and high in fat and protein. They are based on the principle that protein is more filling than carbohydrate, and that having a lower carbohydrate intake produces a more favourable effect on blood pressure [52]. However, LCHF diets have not been found to be more effective for long-term weight reduction than any other diet [50].

Option B is incorrect. Low fat diets, such as the Ornish diet, recommend that 10-20% of daily calories should come from fat, with the remainder coming predominantly from plant-based foods. The proposed advantage of this diet is that the low energy-density plant-based food requires consumption of a large volume food, which should improve satiety [52]. However, low fat diets have not been found to be more effective for long-term weight reduction than any other diet [50].

Option C is incorrect. Mediterranean diets recommend the replacement of saturated fats (such as red meat and dairy) with unsaturated fats (such as olive oil, fish, lean poultry, and nuts), and the consumption of fruit, vegetables, and whole-grains [52]. Compared to LCHF and low-fat diets, Mediterranean diets have been found to have a more favourable effect on glycemic control [55]. However, low fat diets have not been found to be more effective for long-term weight reduction than any other diet [50].

**Option D is correct. Any diet can give similar weight reduction, so long as they provide a net energy deficit, and the diet is maintained [52, 54]. If there is a net energy deficit, neither the relative percentages of the constituent macronutrients, nor the type of diet, affects long-term weight reduction [50, 56]. Perhaps the most important factor when considering a diet is personal preference; encouraging long-term compliance is more important than the type of diet itself [52, 54].**

12. Which of the following is considered to be the most appropriate recommendation when looking at conservative treatment of obesity?

**A. An energy deficit of approximately 600 kcal/day**

B. < 20% of the energy in the diet comes from fat as a macronutrient

C. Weight loss > 1.0 kg/week

D. A diet very low in energy (<800 kcal/day)

**Option A is correct. Ensuring a daily energy deficit is the only method of reducing weight [13]. Conventional hypocaloric diets aim to produce a deficit of 500-750 kcal/day [57], which is considered an appropriate recommendation for conservative treatment for obesity [58].**

Option B is incorrect. Given similar total calories, the relative percentages of the constituent macronutrients in a diet do not affect long-term weight reduction [56].

Option C is incorrect. For most individuals, weight loss > 1.0 kg/week is considered unsafe and increases the risks of developing gallstones and electrolyte abnormalities [58]. Guidelines suggest that aiming for weight loss of between 1-2 lb per week (approximately 0.4-0.9 kg) is safe and achievable [58]. Among individuals with an average BMI of 33, typical weight loss after 6 months while adhering to an energy-deficit diet was 6kg, or an average of 1kg per month (50).

Option D is incorrect. A diet very low in energy (<800 kcal/day) is known as a Very Low Calorie Diet (VLCD). VLCDs typically produce greater short-term weight loss than Low Calorie Diets (LCD), but VLCDs are also associated with greater weight re-gain [59]. In short, VLCDs do not produce greater long-term weight-loss than other dietary approaches. Moreover, without medical supervision, VLCDs are associated with an increased risk of severe complications such as fatal cardiac events [60]. There is some evidence to suggest that VLCDs with a follow-up behavioural program to promote weight loss maintenance, are effective for long-term weight reduction [61]; however, VLCDs are not considered a conservative approach for treatment of obesity.

13. Which of the following is most correct when looking at long-term outcomes of gastric bypass surgery (GBP) as treatment for obesity?

A. Gastric bypass surgery (GBP) improves metabolic risk profile, but not primarily cardiovascular risk

**B. Approximately 15% of patients experience suboptimal weight loss or significant weight re-gain, following gastric bypass surgery (GBP)**

C. Gastric bypass surgery (GBP) does not produce more significant weight loss after two years, compared to lifestyle treatment of obesity

D. Approximately 95% of patients who undergo gastric bypass surgery (GBP) respond well in terms of weight loss

Option A is incorrect. Metabolic risk profile refers to factors involved in Metabolic Syndrome, the name given to a cluster of risk factors that increases the risk for Type II Diabetes, cardiovascular disease, and stroke. Cardiovascular risk refers to risk factors that pertain to cardiovascular risk only. Gastric bypass surgery improves both metabolic risk profile and cardiovascular risk, thus, Option A is incorrect [62, 63].

**Option B is correct. Although there is no universally accepted definition of ‘suboptimal weight loss’ or ‘significant weight re-gain’ in the literature, current estimates suggest that approximately 15% of patients regain a significant portion of their weight following GBP, at long-term follow-up [64].**

Option C is incorrect. Gastric bypass surgery does produce significant weight loss after two years, compared to lifestyle treatment of obesity [65].

Option D is incorrect. As stated in Option B, although there is no agreed-upon definition of what constitutes a patient who ‘respond[s] well in terms of weight loss’, approximately 15% of patients experience significant weight regain or suboptimal weight loss following GBP [64].

14. Which of the following represents the most common complication experienced after gastric bypass surgery (GBP)?

A. Hypertension

B. Dyslipidemia

**C. Low levels of vitamin B12, vitamin D, calcium, and iron**

D. Osteoporosis

Option A is incorrect. Gastric bypass surgery (GBP) is a highly effective treatment for pre-existing hypertension – in other words, hypertension is not a complication of GBP, GBP is a treatment for hypertension [66]. Hypotension, on the other hand, resulting from autonomic insufficiency, is a rare complication of GBP [67].

Option B is incorrect. As in Option A, dyslipidemia is not a complication of GBP; dyslipidemia is a condition that is often resolved by GBP [68].

**Option C is correct. Low levels of vitamin B12, vitamin D, calcium, and iron, are the most common complication experienced after gastric bypass surgery [69]. These deficiencies are caused by an alteration of the normal absorption of nutrients, secondary to bypassing the 1^st^ part of the gastrointestinal tract (duodenum). Dietary supplementation of potential deficiencies is typically begun immediately post-surgery and should be continued indefinitely.**

Option D is incorrect. Although GBP may be associated with reduced bone mineral density (BMD) in the hip and lumbar spine (due to reduced vitamin D deficiency and calcium malabsorption; [70]), a recent meta-analysis did not find that BMD changes produced a clinically significant increase in the incidence of osteoporosis [71].

15. Which of the following is least associated with obesity?

A. Diabetes Mellitus Type II (DM2)

**B. Osteoporosis**

C. Male infertility

D. Non-alcoholic fatty liver disease (NAFLD)

Option A is incorrect. Diabetes Mellitus Type II (DM2) is highly associated with obesity; upwards of 80% of individuals with DM2 are obese [13]. Interestingly, the likelihood of being diagnosed with DM2 increases non-linearly with obesity, from an Odds Ratio (OR) of 1.6 for overweight adults (BMI 25-29.9), to OR 11.6 for adults with class III obesity (BMI > 40) [72].

**Option B is correct. Osteoporosis is least associated with obesity, insofar as the relationship between osteoporosis and obesity is not yet fully understood. Prior to the last 15 years, obesity was thought to be a protective factor against osteoporosis, with many studies reporting a correlation between obesity and increased bone mass [73]. However, more recent studies which accounted for the effect of increased mechanical load on bone mass suggested that obesity may, in fact, be a risk factor for osteoporosis and fragility fractures [74]. Regardless, the science is still not settled – but a reliable association between obesity and osteoporosis cannot yet be drawn.**

Option C is incorrect. Male infertility is associated with obesity. Obesity can cause male infertility resulting from endocrine abnormalities, comorbid conditions, and abnormal spermatogenesis [75].

Option D is incorrect. Non-alcoholic fatty liver disease (NAFLD) is a common cause of chronic liver disease, that may progress to end-stage liver disease [76]. NAFLD is associated with obesity, with the prevalence of NAFLD among obese patients ranging from 57% to 98% [77].

16. What level of physical activity is recommended for individuals with obesity in order to maintain weight loss?

A. 30 min/day moderate intensity

B. 10 min bouts with high intensity, 3 times/week

**C. 45-60 min/day moderate intensity**

D. 30 min high-intensity interval training (HIIT), 3 times/week

Option A is incorrect. 30 min/day of moderate intensity exercise may be sufficient to realize some benefits on cardiovascular health, but it is less than the 45-60 minutes per day typically required to maintain weight loss [78].

Option B is incorrect. 10 min bouts with high intensity 3 times per week (total 30 minutes) is not sufficient to maintain weight loss; guidelines recommend between 150-300 minutes of moderate to vigorous exercise per week in order to maintain weight loss [78].

**Option C is correct. The 2008 physical activity guidelines for Americans suggests that in order to maintain weight loss over a period of 6 months or longer, between 30-60 minutes of moderate to vigorous exercise is required each day for at least 5 days of the week [78]. In the last 10 years, further research has supported the recommendations made in the 2008 report [79, 80].**

Option D is incorrect. Although High-intensity Interval Training (HIIT) is a time-effective method for improving overall cardiovascular health [44], 30 minutes 3 times per week (total 90 minutes) is not sufficient to maintain weight loss.

17. On average, what percentage of individuals who have lost weight through lifestyle changes are able to maintain clinically significant weight loss for at least 1 year?

**A. < 10%**

B. 20%

C. 30%

D. > 40%

**Option A is correct. Clinically significant weight loss is generally regarded as weight loss of between 5-10% of initial weight [38, 40]. Although the differing methodologies make it challenging to compare studies in the literature, lifestyle interventions are typically successful in fewer than 10% of cases [81-83].**

Option B is incorrect.

Option C is incorrect.

Option D is incorrect.

18. Which of the following is most associated with long-term weight loss maintenance?

A. A diet high in carbohydrates (≤ 55% of total energy intake)

B. Exercising at high intensity > 3 times/week

**C. Eating breakfast > 5 days/week**

*D. Self-weighing ≥ 1 times/month*

Option A is incorrect. A diet high in carbohydrates is more likely to promote weight gain, not maintain weight loss, due to the high energy density of carbohydrates and the glycemic response following carbohydrate metabolism [6]; although as stated previously, the only factor associated with weight loss maintenance is net energy deficit, not relative macronutrient contents of the diet [56].

Option B is incorrect. Although exercise is an important factor in long-term weight loss maintenance, the answer choice does not indicate the duration of the high intensity exercise; it is the total duration of exercise that is important in maintaining weight loss (between 150-300 minutes per week of moderate to vigorous exercise is recommended), not the frequency [78].

**Option C is correct. A number of empirical studies and systematic reviews have supported the idea that regular breakfast consumption (RBC) is associated with lower BMI and a greater likelihood of long-term weight loss maintenance [84-88]. However, more recent studies have challenged the relationship between RBC and obesity, with equivocal findings for adults [89]. Others have suggested that the claimed relationship between RBC and obesity may not be sufficiently supported by the strength of the findings in the literature [90]. Regardless, current consensus suggests that RBC is associated with long-term weight loss, although this is currently subject to debate.**

Option D is incorrect. Although self-weighing when included as a component of other behavioural interventions may be effective, there is insufficient evidence to conclude that self-weighing alone is associated with long-term weight loss maintenance [91].

References

1. Carneiro, I.P., et al., *Is Obesity Associated with Altered Energy Expenditure?* Adv Nutr, 2016. **7**(3): p. 476-87.

2. Elbelt, U., et al., *Differences of energy expenditure and physical activity patterns in subjects with various degrees of obesity.* Clin Nutr, 2010. **29**(6): p. 766-72.

3. Faria, S.L., et al., *Metabolic profile of clinically severe obese patients.* Obes Surg, 2012. **22**(8): p. 1257-62.

4. DeLany, J.P., Bray, G. A., Harsha, D. W., & Volaufova, J., *Energy expenditure in African American and white boys and girls in a 2-y follow-up of the Baton Rouge Children’s Study.* The American journal of clinical nutrition, 2004. **79**(2): p. 268-273.

5. Roberts, S.B., Krupa Das, S., & Saltzman, E., *Energy expenditure in obesity.* Am J Clin Nutr, 2004. **79**: p. 181-182.

6. Kahlhofer, J., et al., *Carbohydrate intake and glycemic index affect substrate oxidation during a controlled weight cycle in healthy men.* Eur J Clin Nutr, 2014. **68**(9): p. 1060-6.

7. Lazzer, S., et al., *Relationship between basal metabolic rate, gender, age, and body composition in 8,780 white obese subjects.* Obesity (Silver Spring), 2010. **18**(1): p. 71-8.

8. Fukagawa, N.K., Bandini, L. G., & Young, J. B., *Effect of age on body composition and resting metabolic rate.* American Journal of Physiology-Endocrinology and Metabolism, 1990. **259**(2): p. E233-E238.

9. Verga, S., Buscemi, S., & Caimi, G., *Resting energy expenditure and body composition in morbidly obese, obese and control subjects.* Acta diabetologica, 1994. **31**(1): p. 47-51.

10. Rising, R., Harper, I. T., Fontvielle, A. M., Ferraro, R. T., Spraul, M., & Ravussin, E., *Determinants of total daily energy expenditure: variability in physical activity.* The American journal of clinical nutrition, 1994. **59**(4): p. 800-804.

11. Haynes, A., E. Kemps, and R. Moffitt, *Does trait self-control predict weaker desire for unhealthy stimuli? A lab-based study of unhealthy snack intake.* Personality and Individual Differences, 2016. **89**: p. 69-74.

12. Kuijer, R., et al., *Dieting as a case of behavioural decision making. Does self-control matter?* Appetite, 2008. **51**(3): p. 506-11.

13. Flier J.S., M.-F.E., *Pathobiology of Obesity*. 20e ed. Harrison's Principles of Internal Medicine, ed. F.A.S. Jameson J., Kasper D. L., Hauser S. L., Longo D. L., & Loscalzo J. 2019, New York, NY: McGraw-Hill.

14. Bell, C.G., A.J. Walley, and P. Froguel, *The genetics of human obesity.* Nat Rev Genet, 2005. **6**(3): p. 221-34.

15. Cheskin, L.J., Bartlett, S. J., Zayas, R., Twilley, C. H., Allison, D. B., & Contoreggi, C., *Prescription medications: a modifiable contributor to obesity.* Southern medical journal, 1999. **92**(9): p. 898-904.

16. Morton, G.J., et al., *Central nervous system control of food intake and body weight.* Nature, 2006. **443**(7109): p. 289-95.

17. King, N.A., Caudwell, P., Hopkins, M., Byrne, N. M., Colley, R., Hills, A. P., . . . Blundell, J. E., *Metabolic and behavioral compensatory responses to exercise interventions: barriers to weight loss.* Obesity, 2007. **15**(6): p. 1373-1383.

18. Sumithran, P. and J. Proietto, *The defence of body weight: a physiological basis for weight regain after weight loss.* Clin Sci (Lond), 2013. **124**(4): p. 231-41.

19. Bardia, A., et al., *Diagnosis of obesity by primary care physicians and impact on obesity management.* Mayo Clin Proc, 2007. **82**(8): p. 927-32.

20. Katzow, M., P. Homel, and K. Rhee, *Factors Associated With Documentation of Obesity in the Inpatient Setting.* Hosp Pediatr, 2017. **7**(12): p. 731-738.

21. Shuster, A., et al., *The clinical importance of visceral adiposity: a critical review of methods for visceral adipose tissue analysis.* Br J Radiol, 2012. **85**(1009): p. 1-10.

22. Keys, A., Fidanza, F., Karvonen, M. J., Kimura, N., & Taylor, H. L., *Indices of relative weight and obesity.* Journal of chronic diseases, 1972. **25**(6-7): p. 329-343.

23. Huxley, R., et al., *Body mass index, waist circumference and waist:hip ratio as predictors of cardiovascular risk--a review of the literature.* Eur J Clin Nutr, 2010. **64**(1): p. 16-22.

24. Neovius, M., Y. Linne, and S. Rossner, *BMI, waist-circumference and waist-hip-ratio as diagnostic tests for fatness in adolescents.* Int J Obes (Lond), 2005. **29**(2): p. 163-9.

25. Gill, T., et al., *Body mass index, waist hip ratio, and waist circumference: which measure to classify obesity?* Sozial- und Proventivmedizin/Social and Preventive Medicine, 2003. **48**(3): p. 191-200.

26. de Koning, L., et al., *Waist circumference and waist-to-hip ratio as predictors of cardiovascular events: meta-regression analysis of prospective studies.* Eur Heart J, 2007. **28**(7): p. 850-6.

27. Kiuru, E., Kokki, H., Juvonen, P., Lintula, H., Paajanen, H., Gissler, M., & Eskelinen, M., *The impact of age and sex adjusted body mass index (ISO-BMI) in obese versus non-obese children and adolescents with cholecystectomy.* in vivo, 2014. **28**(4): p. 615-619.

28. Cole, T.J., Bellizzi, M. C., Flegal, K. M., & Dietz, W. H., *Establishing a standard definition for child overweight and obesity worldwide: international survey.* BMJ, 2000. **320**(7244): p. 1240-1243.

29. Flegal, K.M. and C.L. Ogden, *Childhood obesity: are we all speaking the same language?* Adv Nutr, 2011. **2**(2): p. 159S-66S.

30. Nader, P.R., et al., *Identifying risk for obesity in early childhood.* Pediatrics, 2006. **118**(3): p. e594-601.

31. Speiser, P.W., et al., *Childhood obesity.* J Clin Endocrinol Metab, 2005. **90**(3): p. 1871-87.

32. Sharma, A.M. and R.F. Kushner, *A proposed clinical staging system for obesity.* Int J Obes (Lond), 2009. **33**(3): p. 289-95.

33. Kuk, J.L., et al., *Edmonton Obesity Staging System: association with weight history and mortality risk.* Appl Physiol Nutr Metab, 2011. **36**(4): p. 570-6.

34. Padwal, R.S., Pajewski, N. M., Allison, D. B., & Sharma, A. M., *Using the Edmonton obesity staging system to predict mortality in a population-representative cohort of people with overweight and obesity.* CMAJ, 2011. **183**(14): p. E1059-E1066.

35. Chiappetta, S., et al., *The importance of the Edmonton Obesity Staging System in predicting postoperative outcome and 30-day mortality after metabolic surgery.* Surg Obes Relat Dis, 2016. **12**(10): p. 1847-1855.

36. Gill, R.S., S. Karmali, and A.M. Sharma, *The potential role of the Edmonton obesity staging system in determining indications for bariatric surgery.* Obes Surg, 2011. **21**(12): p. 1947-9.

37. Zevin, B., *Personal Communication*. Aug 7, 2019.

38. Douketis, J.D., et al., *Systematic review of long-term weight loss studies in obese adults: clinical significance and applicability to clinical practice.* Int J Obes (Lond), 2005. **29**(10): p. 1153-67.

39. Goodpaster, B.H., Kelley, D. E., Wing, R. R., Meier, A., & Thaete, F. L., *Effects of weight loss on regional fat distribution and insulin sensitivity in obesity.* Diabetes, 1999. **48**(4): p. 839-847.

40. Wing, R.R., & Hill, J. O., *Successful weight loss maintenance.* Annual review of nutrition, 2001. **21**(1): p. 323-341.

41. Katzmarzyk, P.T. and C. Mason, *Prevalence of class I, II and III obesity in Canada.* CMAJ, 2006. **174**(2): p. 156-7.

42. Aune, D., et al., *BMI and all cause mortality: systematic review and non-linear dose-response meta-analysis of 230 cohort studies with 3.74 million deaths among 30.3 million participants.* BMJ, 2016. **353**: p. i2156.

43. Bigaard, J., et al., *Waist circumference and body composition in relation to all-cause mortality in middle-aged men and women.* Int J Obes (Lond), 2005. **29**(7): p. 778-84.

44. Wewege, M., et al., *The effects of high-intensity interval training vs. moderate-intensity continuous training on body composition in overweight and obese adults: a systematic review and meta-analysis.* Obes Rev, 2017. **18**(6): p. 635-646.

45. Foster, C., Farland, C. V., Guidotti, F., Harbin, M., Roberts, B., Schuette, J., . . . Porcari, J. P., *The effects of high intensity interval training vs steady state training on aerobic and anaerobic capacity.* Journal of sports science & medicine, 2015. **14**(4): p. 747.

46. Garcia-Hermoso, A., et al., *Is high-intensity interval training more effective on improving cardiometabolic risk and aerobic capacity than other forms of exercise in overweight and obese youth? A meta-analysis.* Obes Rev, 2016. **17**(6): p. 531-40.

47. Garcia-Hermoso, A., et al., *Concurrent aerobic plus resistance exercise versus aerobic exercise alone to improve health outcomes in paediatric obesity: a systematic review and meta-analysis.* Br J Sports Med, 2018. **52**(3): p. 161-166.

48. Lambers, S., Van Laethem, C., Van Acker, K., & Calders, P., *Influence of combined exercise training on indices of obesity, diabetes and cardiovascular risk in type 2 diabetes patients.* Clin Rehabil, 2008. **22**(6): p. 483-492.

49. Skrypnik, D., et al., *Effects of Endurance and Endurance Strength Training on Body Composition and Physical Capacity in Women with Abdominal Obesity.* Obes Facts, 2015. **8**(3): p. 175-87.

50. Dansinger, M.L., Gleason, J. A., Griffith, J. L., Selker, H. P., & Schaefer, E. J., *Comparison of the Atkins, Ornish, Weight Watchers, and Zone diets for weight loss and heart disease risk reduction: a randomized trial.* JAMA, 2005. **293**(1): p. 43-53.

51. Wu, T., et al., *Long-term effectiveness of diet-plus-exercise interventions vs. diet-only interventions for weight loss: a meta-analysis.* Obes Rev, 2009. **10**(3): p. 313-23.

52. Wadden, T.A., et al., *Lifestyle modification for obesity: new developments in diet, physical activity, and behavior therapy.* Circulation, 2012. **125**(9): p. 1157-70.

53. Donnelly, J.E., et al., *American College of Sports Medicine Position Stand. Appropriate physical activity intervention strategies for weight loss and prevention of weight regain for adults.* Med Sci Sports Exerc, 2009. **41**(2): p. 459-71.

54. Wyatt, H.R., *Update on treatment strategies for obesity.* J Clin Endocrinol Metab, 2013. **98**(4): p. 1299-306.

55. Shai, I., Schwarzfuchs, D., Henkin, Y., Shahar, D. R., Witkow, S., Greenberg, I., . . . Stampfer, M. J., *Weight loss with a low-carbohydrate, Mediterranean, or low-fat diet.* New England Journal of Medicine, 2008. **359**(3): p. 229-241.

56. Sacks, F.M., et al., *Comparison of weight-loss diets with different compositions of fat, protein, and carbohydrates.* N Engl J Med, 2009. **360**(9): p. 859-73.

57. Koliaki, C., et al., *Defining the Optimal Dietary Approach for Safe, Effective and Sustainable Weight Loss in Overweight and Obese Adults.* Healthcare (Basel), 2018. **6**(3).

58. Pi-Sunyer, F.X., Becker, D. M., Bouchard, C., Carleton, R. A., Colditz, G. A., Dietz, W. H., . . . Hansen, B. C., *Clinical guidelines on the identification, evaluation, and treatment of overweight and obesity in adults: Executive summary.* American Journal of Clinical Nutrition, 1998. **68**(4): p. 899-917.

59. Tsai, A.G., & Wadden, T. A., *The evolution of very‐low‐calorie diets: an update and meta‐analysis.* Obesity, 2006. **14**(8): p. 1283-1293.

60. Wadden, T.A., Stunkard, A. J., & Brownell, K. D., *Very low calorie diets: their efficacy, safety, and future.* Annals of Internal Medicine, 1983. **99**(5): p. 675-684.

61. Saris, W.H.M., *Very‐low‐calorie diets and sustained weight loss.* Obesity research, 2001. **9**(S11): p. 295S-301S.

62. Han, T.S. and M.E. Lean, *A clinical perspective of obesity, metabolic syndrome and cardiovascular disease.* JRSM Cardiovasc Dis, 2016. **5**: p. 2048004016633371.

63. Poirier, P., et al., *Bariatric surgery and cardiovascular risk factors: a scientific statement from the American Heart Association.* Circulation, 2011. **123**(15): p. 1683-701.

64. Karmali, S., et al., *Weight recidivism post-bariatric surgery: a systematic review.* Obes Surg, 2013. **23**(11): p. 1922-33.

65. Sjöström, L., Lindroos, A.-K., Peltonen, M., Torgerson, J., Bouchard, C., Carlsson, B., . . . Sjöström, C. D., *Lifestyle, diabetes, and cardiovascular risk factors 10 years after bariatric surgery.* New England Journal of Medicine, 2004. **351**(26): p. 2683-2693.

66. Wilhelm, S.M., J. Young, and P.B. Kale-Pradhan, *Effect of bariatric surgery on hypertension: a meta-analysis.* Ann Pharmacother, 2014. **48**(6): p. 674-82.

67. Billakanty, S.R., Kligman, M. D., Kanjwal, Y. M., Kosinski, D. J., Maly, G. T., Karabin, B., & Grubb, B. P., *New‐Onset Orthostatic Intolerance Following Bariatric Surgery.* Pacing and clinical electrophysiology, 2008. **31**(7): p. 884-888.

68. Spivak, H., et al., *Different effects of bariatric surgical procedures on dyslipidemia: a registry-based analysis.* Surg Obes Relat Dis, 2017. **13**(7): p. 1189-1194.

69. Bloomberg, R.D., Fleishman, A., Nalle, J. E., Herron, D. M., & Kini, S., *Nutritional deficiencies following bariatric surgery: what have we learned.* Obesity surgery, 2005. **15**(2): p. 145-154.

70. Casagrande, D.S., et al., *Changes in bone mineral density in women following 1-year gastric bypass surgery.* Obes Surg, 2012. **22**(8): p. 1287-92.

71. Scibora, L.M., et al., *Examining the link between bariatric surgery, bone loss, and osteoporosis: a review of bone density studies.* Obes Surg, 2012. **22**(4): p. 654-67.

72. Ganz, M.L., Wintfeld, N., Li, Q., Alas, V., Langer, J., & Hammer, M., *The association of body mass index with the risk of type 2 diabetes: a case-control study nested in an electronic health records system in the United States.* Diabetol Metab Syndr, 2014. **6**(1): p. 50.

73. Greco, E.A., et al., *Obesity and Osteoporosis: Is the Paradigm Changing?*, in *Multidisciplinary Approach to Osteoporosis*. 2018. p. 143-152.

74. Zhao, L.-J., Liu, Y.-J., Liu, P.-Y., Hamilton, J., Recker, R. R., & Deng, H.-W. , *Relationship of obesity with osteoporosis.* The Journal of Clinical Endocrinology & Metabolism, 2007. **92**(5): p. 1640-1646.

75. Craig, J.R., et al., *Obesity, male infertility, and the sperm epigenome.* Fertil Steril, 2017. **107**(4): p. 848-859.

76. Angulo, P., *Nonalcoholic fatty liver disease.* New England Journal of Medicine, 2002. **346**(16): p. 1221-1231.

77. Vernon, G., A. Baranova, and Z.M. Younossi, *Systematic review: the epidemiology and natural history of non-alcoholic fatty liver disease and non-alcoholic steatohepatitis in adults.* Aliment Pharmacol Ther, 2011. **34**(3): p. 274-85.

78. Haskell, W.L., & Nelson, M. E., *Physical activity guidelines advisory committee report*. 2008, US Department of Health and Human Services: Washington.

79. Cox, C.E., *Role of Physical Activity for Weight Loss and Weight Maintenance.* Diabetes Spectr, 2017. **30**(3): p. 157-160.

80. Piercy, K.L., et al., *The Physical Activity Guidelines for Americans.* JAMA, 2018. **320**(19): p. 2020-2028.

81. Montesi, L., et al., *Long-term weight loss maintenance for obesity: a multidisciplinary approach.* Diabetes Metab Syndr Obes, 2016. **9**: p. 37-46.

82. Soleymani, T., S. Daniel, and W.T. Garvey, *Weight maintenance: challenges, tools and strategies for primary care physicians.* Obes Rev, 2016. **17**(1): p. 81-93.

83. Wing, R.R., & Phelan, S., *Long-term weight loss maintenance.* The American journal of clinical nutrition, 2005. **82**(1): p. 222S-225S.

84. de la Hunty, A., S. Gibson, and M. Ashwell, *Does regular breakfast cereal consumption help children and adolescents stay slimmer? A systematic review and meta-analysis.* Obes Facts, 2013. **6**(1): p. 70-85.

85. Horikawa, C., et al., *Skipping breakfast and prevalence of overweight and obesity in Asian and Pacific regions: a meta-analysis.* Prev Med, 2011. **53**(4-5): p. 260-7.

86. Laska, M.N., et al., *Longitudinal associations between key dietary behaviors and weight gain over time: transitions through the adolescent years.* Obesity (Silver Spring), 2012. **20**(1): p. 118-25.

87. Mansouri, M., et al., *Breakfast consumption pattern and its association with overweight and obesity among university students: a population-based study.* Eat Weight Disord, 2018.

88. Nurul-Fadhilah, A., et al., *Infrequent breakfast consumption is associated with higher body adiposity and abdominal obesity in Malaysian school-aged adolescents.* PLoS One, 2013. **8**(3): p. e59297.

89. Barr, S.I., L. DiFrancesco, and V.L. Fulgoni, 3rd, *Association of breakfast consumption with body mass index and prevalence of overweight/obesity in a nationally-representative survey of Canadian adults.* Nutr J, 2016. **15**: p. 33.

90. Brown, A.W., M.M. Bohan Brown, and D.B. Allison, *Belief beyond the evidence: using the proposed effect of breakfast on obesity to show 2 practices that distort scientific evidence.* Am J Clin Nutr, 2013. **98**(5): p. 1298-308.

91. Madigan, C.D., et al., *Is self-weighing an effective tool for weight loss: a systematic literature review and meta-analysis.* Int J Behav Nutr Phys Act, 2015. **12**: p. 104.
