# Supplementary material for: A Canadian survey of medical students and undergraduate deans on the management of patients living with obesity
Source: BMC Med Educ. 2022 Jul 21;22:562. doi: 10.1186/s12909-022-03636-9 (PMC9302212; doi:10.1186/s12909-022-03636-9)
Supplement: Supplementary file 6 — Additional file 6. UGME Dean’s Questionnaire. 7-item Questionnaire sent to undergraduate medical school deans of their delegates, assessing curricular teaching of material related to management of patients with obesity. [file 12909_2022_3636_MOESM6_ESM.docx]

**UGME Dean’s Questionnaire**

1. Does your Medical School offer a 4-year or a 3-year MD program?

☐ 4-year program

☐ 3-year program

1. What is the total estimated number of curricular hours spent on teaching obesity-management? (Pre-clerkship and Clerkship combined)
2. What instruction modalities are used to teach obesity-management, as part of the formal UGME curriculum? (Select all that apply. Note that a single form of content delivery may involve multiple instruction modalities listed. e.g. an online *self-directed* learning module)

☐ Lecture

☐ Small group / Tutorial (faculty-directed group learning of 8 or fewer students)

☐ Clinical simulation (e.g. OSCE-style scenarios)

☐ Clinical instruction (on ward / in clinic)

☐ Problem-Based Learning (learning stimulated by encountering problems)

☐ Scheduled Independent Learning

☐ Online Modules / Online Content Delivery

☐ Community-Based Learning

1. The following domains of obesity management are addressed within the UGME curriculum (select all that apply):

☐ Physical Activity Counselling

☐ Nutrition Counselling

☐ Behavioral Therapy

☐ Pharmacotherapy

☐ Surgery

1. Our school has a specific program or non-traditional curricular activity that teaches students about obesity or trains obesity management for clinical practice.

YES / NO

If yes, please describe the curricular activity _________________________

1. Our school offers a dedicated clerkship rotation with a focus on management of patients with obesity. YES / NO
2. During which undergraduate year(s) is management of obesity taught in your school?

*If ‘4-year program’ is selected in question 1: If ‘3-year program’ is selected in question 1:*

☐ Year 1 ☐ Year 1 ☐ Year 2 ☐ Year 2

☐ Year 3 ☐ Year 3

☐ Year 4
